# Non-polyadenylated transcription in embryonic stem cells reveals novel non-coding RNA related to pluripotency and differentiation

**DOI:** 10.1093/nar/gkt316

**Published:** 2013-04-27

**Authors:** Ilana Livyatan, Arigela Harikumar, Malka Nissim-Rafinia, Radharani Duttagupta, Thomas R. Gingeras, Eran Meshorer

**Affiliations:** ^1^Hebrew University of Jerusalem, Jerusalem 91904, Israel, ^2^Affymetrix Inc., Santa Clara, CA 95051, USA and ^3^Cold Spring Harbor Laboratory, New York, NY 11724, USA

## Abstract

The transcriptional landscape in embryonic stem cells (ESCs) and during ESC differentiation has received considerable attention, albeit mostly confined to the polyadenylated fraction of RNA, whereas the non-polyadenylated (NPA) fraction remained largely unexplored. Notwithstanding, the NPA RNA super-family has every potential to participate in the regulation of pluripotency and stem cell fate. We conducted a comprehensive analysis of NPA RNA in ESCs using a combination of whole-genome tiling arrays and deep sequencing technologies. In addition to identifying previously characterized and new non-coding RNA members, we describe a group of novel conserved RNAs (snacRNAs: small NPA conserved), some of which are differentially expressed between ESC and neuronal progenitor cells, providing the first evidence of a novel group of potentially functional NPA RNA involved in the regulation of pluripotency and stem cell fate. We further show that minor spliceosomal small nuclear RNAs, which are NPA, are almost completely absent in ESCs and are upregulated in differentiation. Finally, we show differential processing of the minor intron of the polycomb group gene *Eed*. Our data suggest that NPA RNA, both known and novel, play important roles in ESCs.

## INTRODUCTION

Mature messenger RNA (mRNA) transcripts include a non-templated poly adenosine (polyA) tail at their 3′-ends conferring stability and supporting nuclear export and translation of the RNA transcript to protein (1). As mRNA is the intermediate proxy to protein synthesis and cellular function, in most unbiased genome-wide studies, total RNA is extracted, and polyA is selected via oligo(dT) to enrich for the mRNA subpopulation. This methodology is particularly prevalent in research using state-of-the-art high-throughput sequencing. Hence, the resulting ‘transcriptional landscapes’ of such studies (2–11) are only partial descriptions of genome-wide expression, which may also be comprised of many non-polyadenylated (NPA) RNA transcripts. Comprehensive transcriptome characterization is particularly crucial in a developmental context where cells with the same genetic content undergo drastic widespread changes in gene expression, which regulate and drive the process of differentiation. Embryonic stem cells (ESCs), derived from the blastocyst-stage embryo, were shown to have a permissive transcriptional program throughout their genome with evidence of transcription from larger portions of the genome when compared with differentiated cells (12,13), which potentially underlies their pluripotent nature. But, like the aforementioned studies, this characterization, which was recently challenged (14), relies mainly on studies of the polyadenylated (PA) RNA fraction (6,12), while the NPA RNA remained largely unexplored.

Unlike the PA fraction, the NPA fraction has diverse RNA characteristics. One group is actually protein-coding, despite the lack of a polyA tail: the replication dependent, or canonical histone genes, which encode for histone proteins, are intensively transcribed in S-phase of the cell cycle and are not subject to standard RNA processing mechanisms such as polyadenylation and RNA splicing. Instead, they are stabilized by a stem-loop binding protein, which binds to a conserved stem-loop element at their 3′-ends (15). As histone proteins are the fundamental building blocks of basic and higher-order chromatin structure, their gene expression levels and regulation is particularly interesting in ESCs where chromatin is characterized as open and dynamic (16,17).

Conversely, there are many non-coding RNAs (ncRNA), which do not undergo translation, but function as RNA transcripts. Long ribosomal RNA (rRNA) and transfer RNA (tRNA) act as core functional elements in the process of mRNA translation. Short ncRNAs include small nuclear RNAs (snRNAs) that participate in the RNA-splicing process as part of the core spliceosomal machinery; small nucleolar RNAs (snoRNA) and small Cajal Body RNAs that have roles in the post-transcriptional processing of rRNA and snRNAs; piwi-interacting RNAs (piRNAs) that are associated with transcriptional silencing of retrotransposons; and micro RNAs (miRNAs) that regulate transcriptional levels and translation of target mRNAs. An additional handful of other singleton NPA ncRNA examples carry out critical cellular functions such as Telomerase, which lengthens telomeres in rapidly dividing cells such as ESCs and in many transformed malignant cell types. Some of these ncRNA families may be initially transcribed with a PolyA tail, but undergo regulated RNA processing leading to their functional, NPA form (18,19). Some are also transcribed by polymerases other than RNA polymerase II (20–22), which may indicate different methods of regulation and stabilization and may also require different methods of detection.

Still, in comparison with the standard protein-coding genes, few ncRNA genes have fully characterized function; yet, it has been hypothesized that ncRNA may partially explain differences in complexity among organisms (23) and could possibly carry out key roles in differentiation and development processes.

In our study, we divided total RNA from murine ESCs and neuronal progenitor cells (NPCs) derived from ESCs by *in vitro* differentiation (12) into an enriched PA (PolyA+) fraction and an NPA fraction. We analyzed both fractions separately on both whole-genome tiling microarrays, enabling us to separate signal from experimental noise, and the NPA fraction further with high-throughput sequencing technologies, taking advantage of the complementarities of the two methods (24). We find that the NPA RNA fraction is enriched with non-coding transcription. Matching our results with existing RNA databases (i.e. fRNAdb), we were able to correct or improve the annotation of several known ncRNA transcripts. Importantly, we identify novel highly conserved ncRNA genes (‘snacRNAs’, small NPA conserved RNAs), some of which are significantly differentially expressed between ESCs and NPCs, indicating a potential role in stem cell maintenance, pluripotency and/or cell differentiation. We show a potential regulatory link between the NPA ncRNA of the minor spliceosome and the expression levels of pluripotency and developmental genes and show that at least one of the ncRNAs is essential. Additionally, we recognize a unique epigenetic signature for replication-dependent histone genes, which we confirm with our analysis as the only eukaryotic, NPA, protein-coding genes.

## MATERIALS AND METHODS

### Cells

Mouse R1 male ESCs were grown and differentiated into 7-day ESC-derived NPCs (12,16): ESCs were grown on a feeder layer of Mitomycin C treated MEFs on 0.2% gelatin-coated cell culture dishes. Culture medium consisted of Dulbecco’s modified Eagle’s medium, 15% ES-grade fetal calf serum (Biological Industries), 1 mM sodium pyruvate (Biological Industries), 0.1 mM non-essential amino acids (Biological Industries), 0.1 mM β-mercaptoethanol (Sigma), 100 U/ml penicillin and 0.1 mg/ml streptomycin and 1000 U/ml leukemia inhibitory factor. ESCs were maintained at 37°C, 5% CO2 and fed with fresh media every day. For differentiation, ESCs were plated on bacterial culture dishes without leukemia inhibitory factor for 4 days to allow for embryoid body formation. Embryoid bodies were replated on tissue culture plates coated with poly-L-ornithine (Sigma) and fibronectin (R&D systems), in Dulbecco’s modified Eagle’s medium/F12 medium (Sigma) supplemented with Insulin/Transferrin/Selenium (ITS) (5 mg/ml insulin, 50 mg/ml transferrin, 30 nM selenium chloride, Gibco) and 5 mg/ml fibronectin. Media was changed on day 6 and NPCs gathered on day 7. The R1 ESC differentiation system has previously been extensively characterized (16,25).

### RNA extraction and fractionation

We extracted total RNA from undifferentiated ESCs and 7 day NPCs. Total RNA was divided into poly(A+) and poly(A−) enriched fractions using three rounds of a modified version of Oligotex extraction (Qiagen). Briefly, after the hybridization of the total RNA to oligo(dT) beads, supernatants were saved rather than discarded and then subjected to isopropanol-ethanol extraction to obtain the Poly(A−) fraction. Enrichment of PA transcripts in the poly(A)+ RNA and of NPA transcripts in the poly(A)− RNA was assessed using primers specific for *Gapdh* and *Oct4* for the PA fraction and of histone H4 and *Snord32* for the NPA fraction. We ensured that the NPA transcripts cannot be amplified when reverse transcribed with oligo-d(T) primers, only when random primers were used.

### Microarray hybridization

Samples were prepared and labeled as described (26). Briefly, double-stranded cDNA was prepared from poly(A−) or poly(A+) RNA, and 2 mg of double-stranded cDNA was labeled and hybridized to the GeneChip Mouse Tiling Array 1.0R array set (Affymetrix) as described (12). Graph files depicting normalized fluorescence levels per tiled probe were generated via the Affymetrix pipeline (12). Graph files were translated from mouse genome assembly 33 (mm5) to assembly 37 (mm9) using UCSC liftover.

### Microarray analysis

Tiling array probes were subjected to two main criteria: normalized fluorescent levels in NPA samples (i) above noise threshold and (ii) greater than the normalized fluorescent levels in the reciprocal PA samples. Only probes that fulfilled these criteria in each biological replicate separately were called ‘positive’ and used in transfrag analysis. Transfrags (continuous fragments of transcribed units) were assembled from these ‘positive’ probes as described in Kampa *et al.* (8). Briefly, transfrags are assembled from positive probes by meeting two additional criteria: (i) a maximum gap between positive probes (maxgap) and (ii) a minimum length of adjacent positive probes (minrun). We tested a number of maxgap and minrun values and finally selected minrun = 50 and maxgap = 40, which acheived a detection rate of 95% for histone genes (positive control). Transfrags that contained less than three positive probes (∼90 bp in genomic space) were discounted from further analysis. Transfrags were translated to the mm9 genome version using UCSC liftover. Those that intersected with regions from the mm9 Repeat Masker table were filtered out.

### Genic region analysis

Refseq mm9 genes were parsed into exonic and intronic regions. Transfrags were considered exonic if completely or partially intersecting exonic regions, intronic if entirely intersecting intronic regions and otherwise intergenic. For normalization purposes, the total exonic and intronic proportions of the genome were calculated based on the sums of all non-redundant Refseq exons’ or introns’ sizes divided by total genome size, which was inferred from the chromosome lengths in the UCSC ChromInfo table. Significance of enrichment was calculated using Fisher’s exact test. A 5′ or 3′ bias of transfrag overlap within genic regions was measured by dividing exons into three categories: 5′ exons are exons located upstream of the median gene length, 3′ exons located downstream of the median gene length or exons of genes that harbor only one exon.

### Gene expression profile analysis

An expression profile of ‘on’ or ‘off’ was determined for each Refseq gene as described in Bertone *et al.* (27). Briefly, exonic probes consistently above median probe intensity in all biological replicates were assigned a value of 1, otherwise their value was zero. For each gene, the non-parametric sign test was applied with *P* = 1/2 and *n* = total number of exonic probes in the gene region. Genes with *P*-values < 0.05 were considered ‘on’, as this represents a higher than expected number of probes above median probe intensity. Otherwise, genes were assigned an ‘off’ profile. The mean fluorescence intensity level of all exonic probes within a gene was used as a measure of gene expression level.

### RNA library preparation

Library was constructed from 2 µg RNA starting material using the SOLiD™ Whole Transcriptome Analysis Kit (PN 4425680). Briefly, rRNA was removed using one round Life technologies’ RiboMinus Eukaryote kit (A1083708). RNA was fragmented by RNaseIII (10 min, 37°C). Adaptors were hybridized and ligated directly to the fragmented RNA, and reverse transcription was performed on the ligation product. Size selection was done on 4% agarose gels: 150–250 bp fragments were excised and purified using Qiagen MinElute Gel extraction Kit (28604). Library was then amplified by 15 cycles of polymerase chain reaction (PCR). In all, 1400 pmole of each library was amplified on magnetic beads (ePCR) and sequenced on 1/8 sequencing slide, on SOLiD 3 machine.

### RNA-seq analysis

Reads of 50 nt were aligned to the mm9 genome using Bowtie (28). Seed sequence length was set at 45 nt, which had median phred quality score consistently above 15 (base-calling accuracy >97%). A maximum of two mismatches in the seed were allowed. Uniqueness of alignment was not enforced, and reads that mapped to more than one genomic location were dealt with via the ‘best’ option, which chooses the best mapping based on lowest number of mismatches and highest read quality scores. To be comparative with the tiling array platform, we filtered out reads that mapped to the mitochondrial chromosome, to an index of murine ribosomal sequences, or intersected with Repeat Masker regions including both rRNA and tRNA gene regions. For viewing purposes, wiggle plots were generated from the Bowtie generated .sam alignment files and uploaded to the Affymetrix Integrated Genome Browser (IGB) Viewer or translated into .bed file format and uploaded as a UCSC track (see ‘Data Access’ section). In-house MATLAB functions were used to calculate and plot the strand-specific reads per kilo base per million (RPKM) of genomic regions based on the following equation: 10^9^ × number of reads in region/(length of region × total number of reads in the sample). Of 30 million reads, >9 million reads were mapped for each ESCs and NPCs sample. Boxplot quality scores for the first 1 million reads for both ESCs and NPCs are shown (Supplementary Figure S13).

### Northern blot

Ten micrograms of total RNA in loading buffer was denatured at 65°C for 15 min and loaded onto 15% PAGE containing 7 M urea. The small RNAs were then transferred to a Nylon membrane and immobilized under UV. The membrane was pre-hybridized for 2 h at 65°C in prehybridization buffer (7% SDS, 6 × saline-sodium citrate (SSC)). Hybridization was carried out overnight at 42° by using [α-32P] labeled DNA probe. The membrane was then washed three times in 3× SSC/0.1% SDS at room temperature and exposed to a PhosphorImager screen. U6 was used as loading control. Oligonucleotide sequences used in this study were as follows: m-snora61: 5′-AGTCTGGCCTGCTGCTCTAC-3′, U6: 5′-GCCATGCTAATCTTCTCTGTATC-3′.

### Reverse transcriptase-PCR

RNA was extracted with RNeasy (Qiagen). RNase-free DNase (Qiagen) was applied to the column to remove traces of genomic DNA according to protocol. Reverse transcription (RT) was performed on 1 µg of total RNA with High capacity cDNA reverse transcription kit (Applied Biosystems) with random hexamer primers or with oligo-dT_15_ primer (Promega). PCR reactions were carried out in 20 µl volumes using 2× DreamTaq Green PCR Master Mix (Fermentas) and 1 µl of 1:10 diluted cDNA template. Parallel samples without RT enzyme were prepared to detect DNA contamination. PCR conditions were 94°C for 5 min, followed by 30 cycles of 94°C for 30 s, 60°C for 20 s, 72°C for 30 s. Primer sequences for validations can be found in Supplementary Table S1.

### Quantitative PCR

Quantitative (qRT-PCR) reactions were performed with ABI PRISM 7900HT Sequence Detection System in 15 µl volume containing 7.5 µl Power SYBR Green PCR Master Mix (Applied Biosystems). PCR conditions were 50°C for 2 min, 95°C for 10 min, followed by 40 cycles of 95°C for 15 s, 60°C for 1 min. Melting curve analysis was used to confirm the homogeneity of each PCR product. Standard curves including 5 serial dilutions were carried out for each primer pair. Each experimental sample was tested in duplicate for three biological replicates of ESCs and ESC-derived NPCs.

### Knockdown experiments

Antisense oligo (ASO) specific to *Snora61* was designed according to its secondary structure obtained with Kinefold (29) based on the full length, ∼120 nt, which includes the original mouse annotation and the newer NPA transfrag portion, which is homologous to the human *Snora61* sequence. Oligos were targeted at ssRNA regions of the newly detected 5′ portion overlapped by the NPA transfrag. 2′-*O*-Methyl modified RNA/DNA chimera oligos were used in this study. The sequences of the ASOs are as follows (stars represent the phosphorothioate bond between nucleotides):

GFP ASO: 5- mU*mC*mA* mC*mC*T* T*C*A* C*C*C* T*C*T* mC*mC*mA* mC*mU -3
Snora61-ASO: 5- mC*mC*mA* mA*mG*A* C*C*A* G*C*G* T*C*A* mA*mG*mA* mU*mC -3

R1 cells were seeded in mouse ES cell media 1 day before transfection in a 6-well plate. Cells were transfected with 100 nM ASO by using Lipofectamine-2000 according to manufacturer’s protocol. After 4–6 h post-transfection, media was replaced with fresh ES cell media and cell growth conditions were maintained for up to 48 h.

### U12 intron analysis

Expression levels of mRNA and pre-mRNA of 5 U12 intron-containing genes were measured in ESCs and NPCs using qPCR. The geometric mean of Ct values of three genes, *Gapdh*, *18S* and Lamin B was used to normalize sample Ct values. Splicing efficiency values were calculated as the ratio of (normalized) spliced mRNA to unspliced pre-mRNA. Primers for *Drap1*, *Gars* and *Psmc4* were designed based on Pessa *et al.* (30). *Phb2* and *Eed* primers were designed independently. Briefly, two primer pairs were used for mRNA measurements: one set spanning the U12 intron and the second set spanning a nearby U2 intron for each gene. When compatible with qPCR efficiency, one primer included exon junction sequence, otherwise primers were entirely localized within spanning exons. Similarly, two primer pairs were used for pre-mRNA measurements for the same U12 and U2 introns. Where permitting, one primer included the exon–intron boundary sequence and the other was exonic. Otherwise, one primer was exonic and its pair was intronic. Primer sequences can be found in Supplementary Table S2. Semi-quantitative RT-PCR was performed as described in ‘Reverse transcriptase-PCR’ section except that 31 cycles and 28 cycles were used to amplify U12 and U11 transcripts, respectively. Products were run on a 1.6% agarose gel with 50-bp ladder to ensure specificity of product.

### fRNAdb analysis

NPA transfrag sequences from ESCs and NPCs were BLAST-ed against all the sequences in the fRNAdb (31) with the ‘Expect’ parameter set at 0.01 and the penalties for opening and extending gaps at 5 and 2, respectively. An NPA transfrag was considered to successfully align to an fRNAdb sequence if it fulfilled the BLAST criteria and shared at least a 95% identity with it. Additionally sequences were queried via the Infernal tool (32) on the Rfam (33) website, and the results can be viewed in Supplementary Table S3.

### Gene ontology

DAVID (34) was used to establish gene ontology annotations and clustering for given sets of genes. Only Benjamini–Hochberg corrected *P*-values of <0.05 were considered significant.

### Conservation analysis

PhastCons (35) scores from 30-way alignment of placental mammals stored in the phastCons30way table in UCSC were used to calculate mean conservation levels of genomic regions. To assess significance of conservation for intergenic NPA transfrags, a random set of the same number intergenic loci with the same lengths as the NPA transfrags were generated, and the distributions of the conservation scores were compared between the NPA transfrags and the random set. *P*-values were obtained by applying the Kolmogorov–Smirnov test. This process was repeated 50 times to ensure robustness of outcome.

### Epigenetics analysis

Aligned sequencing reads from chromatin immunoprecipitation experiments performed on various histone modifications in ESCs and NPCs (36) were used in this analysis. SeqMiner 1.2 (37) was used to K-means cluster and view the distribution of these aligned reads in the 10 000-nt vicinity of center-aligned genomic regions belonging to intergenic snacRNAs and histone genes. Value for number of clusters for K-means clustering was set at 6 after trying a few different numbers. Density plots of histone modifications per cluster were created in Excel using binned read density values obtained from SeqMiner analysis.

## Data access

The data have been submitted to Gene Expression Omnibus (GEO) under accession number GSE37338.

UCSC custom tracks representing the validated NPA transfrags and mapped sequencing reads can be easily uploaded in the genome browser by copy-pasting lines from Supplementary File S1.

## RESULTS

### Identification of NPA transcription

**Figure 1.**
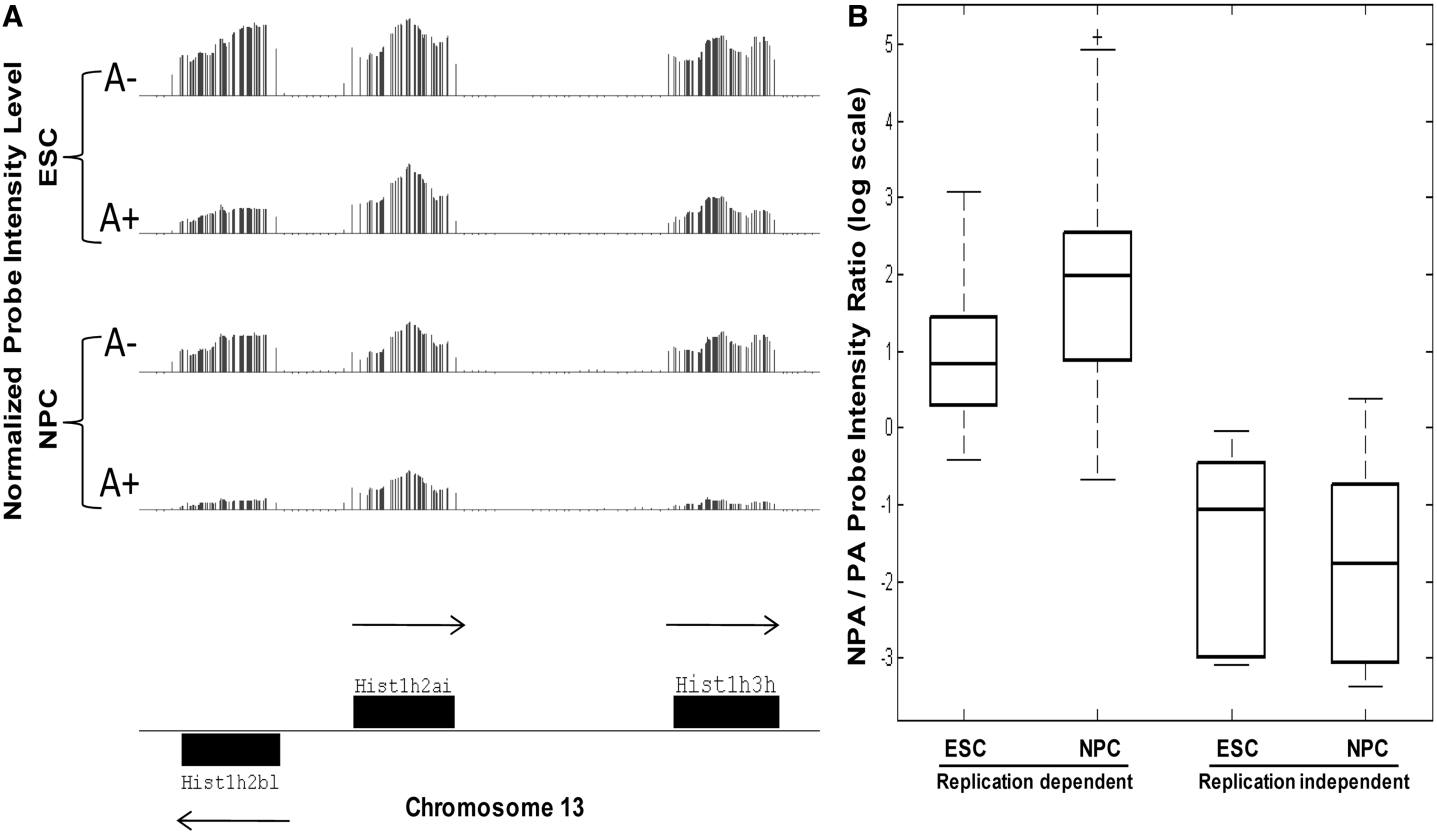
Tiling array detects NPA replication-dependent histone genes. (**A**) Normalized tiling array probe intensities corresponding to a replication-dependent histone gene cluster on mouse chromosome 13. Each vertical line represents the normalized intensity level of one tiled probe from this genomic region. Shown are probe intensities from one biological repeat of NPA (A−) and polyadenylated (A+) ESC (top) versus NPC (bottom). Probe intensities are higher in the A− than the A+ samples in both cell types (**B**) Boxplot of histone genes’ NPA to PA average intensity ratios. Average intensity level for each histone gene was calculated by averaging the intensities of all the tiled probes within the exonic region over all biological repeats. The NPA/PA ratios of the replication-dependent histone genes is greater than 1, whereas the polyadenylated replication-independent (variant) histones display lower ratios.

Genome-wide, PA and NPA transcriptomes were obtained for murine ESCs and ESC-derived NPCs: RNA was extracted from these cell types in three biological repeats, and then fractionated into PA and NPA fractions via oligo(dT) columns. Each fraction was independently hybridized to a repeat-masked Affymetrix whole mouse genome tiling array at 30-bp resolution (see ‘Materials and Methods’ section). The normalized intensity signal from the NPA sample clearly correlates with exons of a replication-dependent histone cluster in both cell types, which have been extensively characterized as the only protein coding genes in the eukaryotic genome to encode for mRNAs that do not contain a poly A tail at their 3′-end (15) (Figure 1A, rows 1 and 3). For these genes, intensity peaks correlating to the histone gene cluster exons can be detected in the PA sample as well (Figure 1A, rows 2 and 4), but at lower levels than the NPA signals. The bulk of the NPA/PA signal intensity ratios distribution for all replication-dependent histones is consistently >1 with a dynamic range of up to >100-fold (Figure 1B). Replication-independent histone genes (variant histones) show the opposite trend (Figure 1B) and are expressed as PA RNA.

Although some studies have deemed expressed regions that show both PA and NPA transcription signals as ‘bimorphic’ (producing both transcripts with polyA tails and without polyA tails) (5,38,39), there is strong biological evidence that canonical replication-dependent histone transcripts do not undergo polyadenylation in any biological circumstance (40). The PA signal for the canonical histone genes is more likely the result of contamination of the PA sample with non-polyadenlyated transcripts, as the RNA fractionation methodology provides enriched PA and NPA fractions, but does not guarantee exclusivity. An accurate analysis of NPA transcription must take the PA transcriptional levels into account in effort to clean out this contamination factor. By analyzing PA and NPA samples side by side, we were able to account for the PA contamination in the NPA samples. To this end, probes that were both above a minimal noise threshold and had higher NPA over PA intensity levels were considered in subsequent analysis to define regions of NPA transcription. These criteria were applied to each biological repeat separately to ensure robustness of the outcome. Positive probes that passed the filter were arranged into ‘transfrags’ (8,41). Several minrun and maxgap criteria were tested and minrun of 50 and maxgap of 40 produced the highest detection rate of canonical histones (>95%) in ESC and NPC samples. Transfrags represented by less than three positive probes were discounted from further analyses to minimize false positive detection. Transfrag coordinates were translated to the most updated mm9 mouse genome version and all subsequent analyses carried out in that realm unless otherwise indicated. To be consistent with original repeat-masked Affymetrix tiling array probe design, an additional round of repeat masking was applied to the updated NPA transfrags based on the mm9 Repeat Masker regions. After processing, a total of 2382 NPA transfrags were obtained from the ESC samples and 3859 NPA ranges from the NPC samples with a highly similar mean/median length of 135/89 nt and 125/90 nt, respectively. We validated the tiling array results using RT-PCR on randomly selected transfrags from both cell types (Supplementary Figure S1), obtaining validation rates of 83 and 75% for ESCs and NPCs, respectively.

### NPA transcription has an opposing trend to that of PA transcription

**Figure 2.**
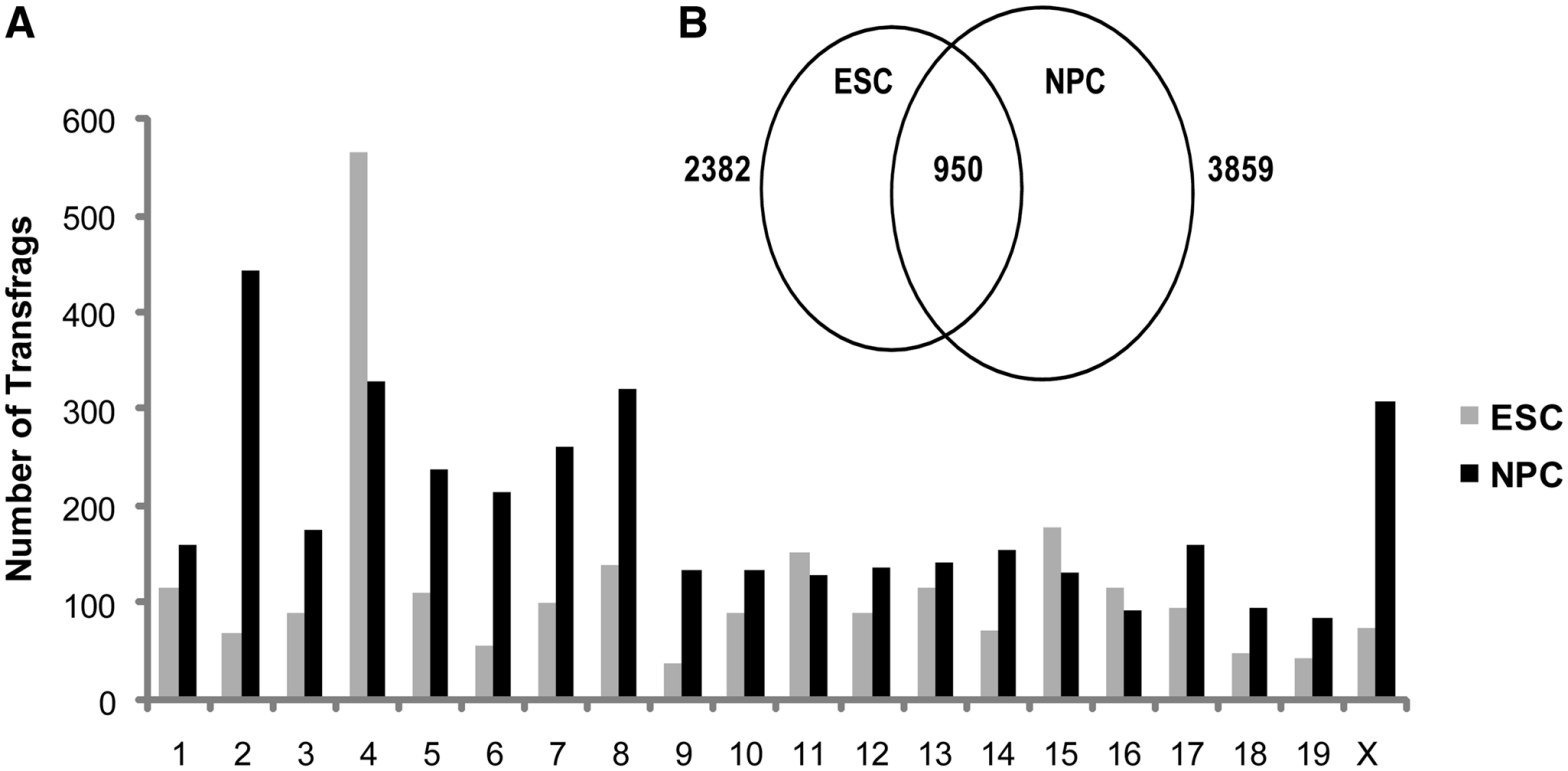
NPA transcription increases during ESC differentiation. (**A**) The number of NPA transfrags (see ‘Materials and Methods’ section) per chromosome in ESCs (gray) and NPCs (black). (**B**) Venn diagram of NPA transfrags from ESCs (left) and NPCs (right). Nine hundred and fifty transfrags overlap, whereas the majority are cell type specific with a large excess found in the differentiated NPC cell type (2909 versus 1432 unique NPA transcripts in NPCs and ESCs, respectively).

Surprisingly, the trend of genome-wide NPA transcription stands in sharp contrast to the global hyperactive nature of PA transcription in ESCs versus differentiated cell types (12,13). Both numbers of positive NPA probes and NPA transfrags are higher in NPCs than ESCs, consistently over most mouse chromosomes (Figure 2A, Supplementary Figure S2). The intersection of transfrag intervals from the two groups showed almost 1000 NPA regions common to the two cell types, but many more are cell-type specific with a clear excess of ranges in NPCs (Figure 2B).

### Characterization of protein-coding NPA transcription

**Figure 3.**
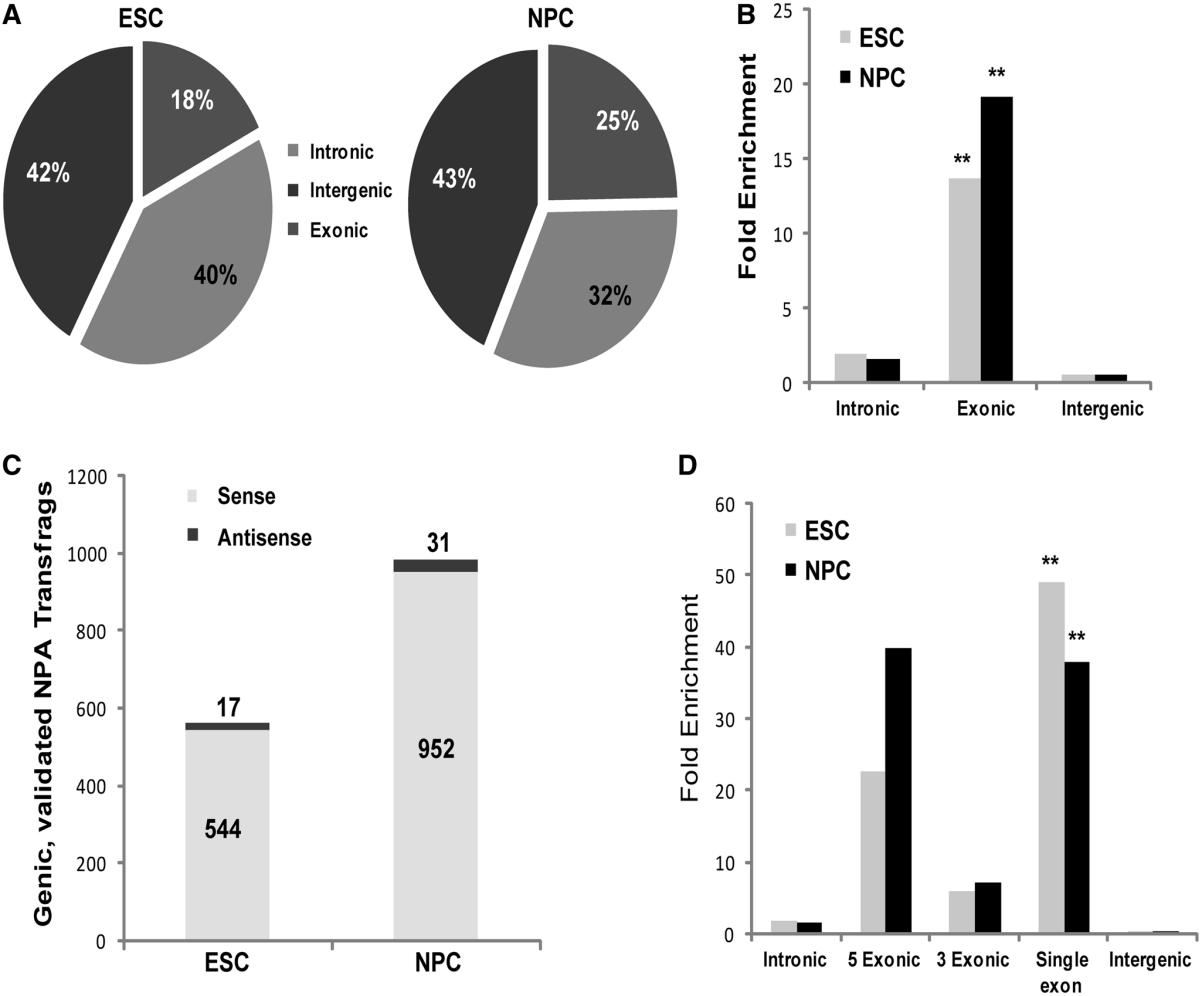
NPA transcription is enriched at protein-coding exons. (**A**) Distribution of number of transfrags in ESCs (left) and NPCs (right) overlapping Refseq exons, introns and intergenic regions. (**B**) Fold enrichment of transfrag overlap with genomic regions in ESCs (gray) and NPCs (black) was calculated after normalizing to their proportional representation in the genome. Overlap with Refseq exons is significantly enriched in both ESCs and NPCs (double asterisk indicates *P* = 0, Fisher’s exact test). (**C**) Orientation of overlap with Refseq genes is in the sense direction. Transfrags that overlapped genic regions (‘genic’) and mapped reads from the strand-specific RNA-seq experiment (‘validated’) were used in this analysis. Transfrags’ directionalities were inferred from the strand-specific RNA-seq mapped reads that overlapped them. Only 3% of transfrags were in antisense orientation to overlapping genes. (**D**) Fold enrichment of transfrag overlap with intronic, intergenic and exonic regions (5′ exons, 3′ exons and exons from single exon genes). Note overlap bias of 5′ over 3′ exons. Overlap with single exon genes, comprised mostly of histone genes, is also enriched.

To explore the relationship between genome-wide NPA and PA transcription, we assessed the locations of NPA transfrags relative to known PA genes. As expected, most NPA transfrags from both cell types overlapped intronic and intergenic regions, as these comprise the majority of the genome (Figure 3A and B). However, after normalization according to the relative proportions of the different regions in the genome (see ‘Materials and Methods’ section), we found a strong enrichment in overlap of NPA transfrags with exonic regions of annotated PA genes (‘exonic’ transfrags) (*P* = 0, Fisher’s exact test) (Figure 3B).

The significant overlap with exons of known PA genes piqued our curiosity as to whether the NPA transfrag transcription was coming from the same strand as the overlapping host gene or in the antisense orientation to it. Widespread antisense transcription has been detected in mammalian organisms (42–45), and many mechanisms of transcriptional repression in eukaryotic cells rely on base pairing between sense and antisense RNA transcripts (46,47). A proposed repressive regulatory role of NPA transcripts on PA transcripts could be mediated via sense–antisense pairing with PA mRNAs. As the tiling array data are insufficient for distinguishing the strand-specificity of NPA transcription, we performed strand-specific RNA-seq on NPA RNA fractions from ESCs and NPCs using ABI’s SOLiD technology. To isolate the NPA fraction, we first eliminated the mRNA fraction using oligo(dT) columns and then eliminated the rRNA with Ribominus (see ‘Materials and Methods’ section). We validated the enrichment of NPA transcripts using primers specific for PA genes (*Gapdh*, *Oct4*), for the NPA histone H4 mRNAs and for *Snord32*. In all cases, we found a significant NPA enrichment spanning an order of magnitude. We also verified that the NPA transcripts do not amplify (by RT-PCR) when reverse transcribed with oligo-d(T) primers, but only when random primers are used.

Approximately 30% of NPA transfrags detected by the tiling array contained at least one mapped read. We considered these NPA transfrags as ‘validated’ by the RNA-seq experiment, as these transfrags already passed stringent criteria in the tiling array analysis, and the RNA-seq analysis was also performed under stringent conditions (i.e. lower coverage and stringent mapping conditions) (Supplementary Table S3). We inferred the orientation of each validated NPA transfrag according to the overlapping mapped reads’ strands and compared the direction of the NPA transcription with that of the host gene (inferred from the annotation). Surprisingly, only 3% of validated NPA transfrags were antisense to their overlapping host genes (Figure 3C). Of these potentially antisense transfrags, a handful seems to stem from incorrect strand annotation of PA genes in the UCSC refGene table, e.g. *Hist1h2bm*, *Hist1h2bb*, *Rpph1*. Others overlap genomic locales that contain more than one PA gene and could, therefore, have been mis-assigned to the incorrect host gene, e.g. *Airn* and *Igf2r*. This contradicted our hypothesis that the exonic NPA transfrags represent antisense transcription.

So what do these NPA transfrags, which overlap exons of PA genes in the same (sense) orientation to the host gene, represent? We noticed that, in most cases, exonic NPA transfrags overlap only a subset of the exons within a protein-coding gene with an apparent bias toward exons at the 5′-end. We validated the perceived bias by categorizing PA Refseq exons into three subcategories: (i) 5′ exon of a multi-exon protein-coding transcript, (ii) 3′ exon of a multi-exon protein-coding transcript, and (iii) exon of a protein-coding transcript with an exon count of 1 (Single Exon). The enrichment of overlap with 5′ exons is three times greater than that of 3′ exons in ESCs and over five times greater in NPCs (Figure 3D, Supplementary Figure S3A). From this observation, it seems unlikely that these exonic NPA transfrags represent full-length mRNA (with none or short polyA tails), as equal representation of all exons would be expected in this case. There was no corresponding enrichment in 5′ intronic transfrags over 3′ intronic ones (data not shown), suggesting that these exonic transfrags represent partially degraded mature mRNAs and not nascent pre-mRNA transcripts. Additionally, the tiling array probe intensity profile over a multi-exonic gene shows clear correlation of high probe intensities with annotated exons, whereas probe levels representing intronic regions are mostly at noise level (Supplementary Figure S3B). This suggests that the NPA RNA fraction as obtained by oligo-d(T) fractionation does not contain a high percentage of nascent RNA transcripts, although this option cannot be completely ruled out, as pre-mRNA splicing can occur co-transcriptionally (48). As expected, the ‘Single Exon’ category is significantly enriched in both cell types (*P* < 10^−^^10^, Fisher’s exact test) (Figure 3D), as the intron-less, NPA and replication-dependent histone genes comprise most of that category.

### Characterization of non-coding NPA RNA

**Figure 4.**
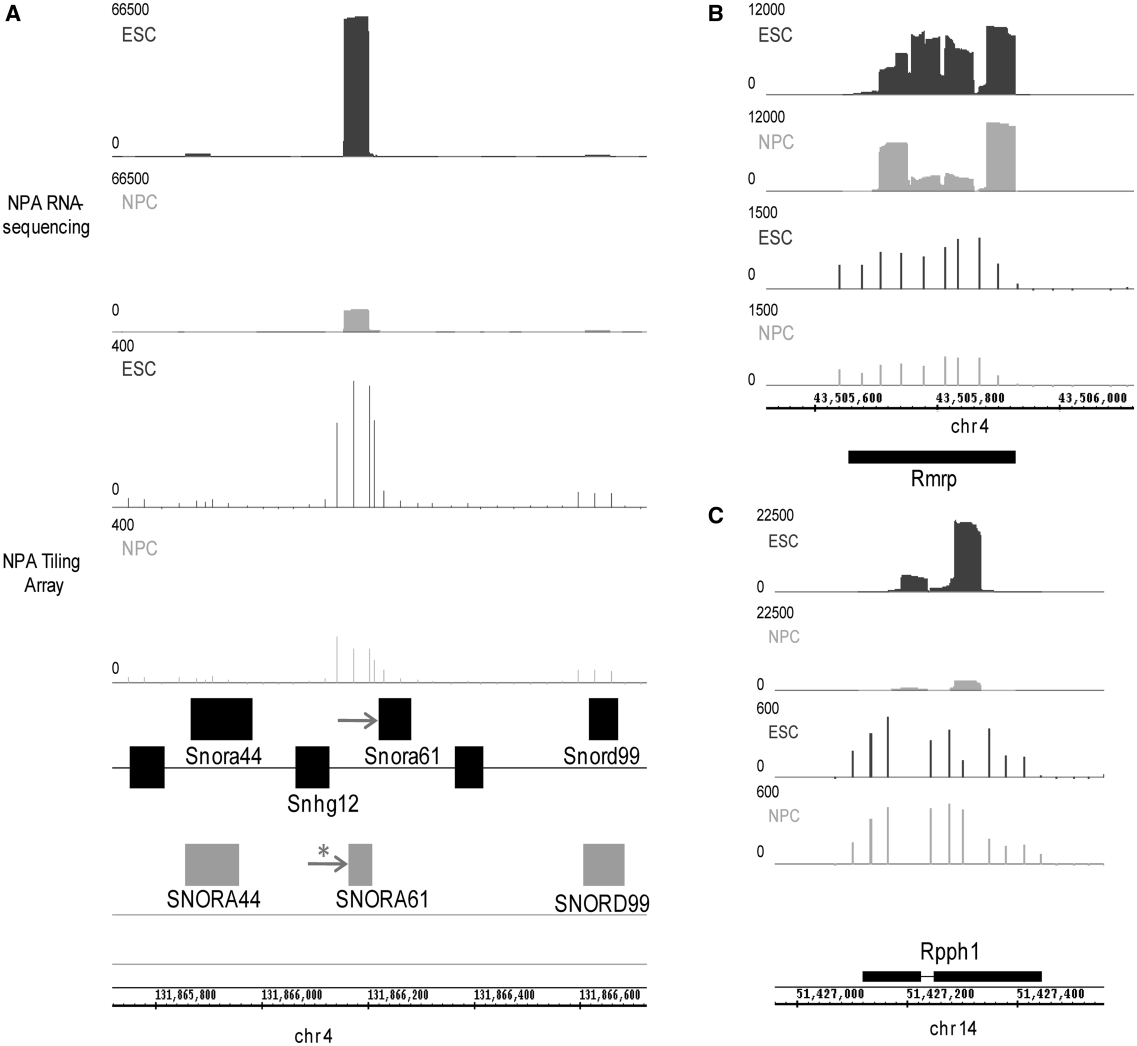
NPA transcriptome contains ncRNA transcripts. (**A**) Read density plots (top two rows) and tiling array probe intensities (rows 3 and 4) from ESCs (dark gray) and NPCs (light gray) around the genomic region of Snhg12 gene on chromosome 4. Black boxes on bottom portray some internal exons of the Snhg12 gene and the non-coding snoRNA genes residing within its introns. Gray boxes show exons of human non-coding snoRNAs aligned to their homologous regions in the mouse genome (taken from non-mouse Refseq track). Peaks of mapped read densities and probe intensities correspond to intronic sequence with high sequence similarity to human Snora61 (starred arrow). Mouse Snora61 ncRNA gene of provisional status (plain arrow) has far less-sequencing representation. Sequence alignment and NPA transcription status better define the genomic location and annotation of mouse Snora61. This transfrag has a maximum number of reads per transfrag of >60 000 in the ESC sample, which drops almost 6-fold during differentiation to NPCs. (**B** and **C**) Read density plots (top two rows) and tiling array probe intensities (rows 3 and 4) from ESCs (dark gray) and NPCs (light gray) around the genomic regions of non-coding *Rmrp* and *Rpph1* genes, respectively. Chromosomal mm9 coordinates shown at the bottom.

A small, 70 nt, intronic region on chromosome 4 had the highest number of reads per transfrag of 66 236 in the ESC sample, accounting for 1.5% of all mapped reads and corresponding to almost 1000× coverage. The number of reads on the opposite strand for this transfrag was zero, indicating the highly significant strand-specific nature of the data (Figure 4A). This NPA transfrag has high sequence similarity with Human *SNORA61* (starred arrow), an snoRNA gene, and resides within the intron of the *Snhg12* (snoRNA hostgene 12) gene. This transfrag location is close to the mouse *Snora61* gene of ‘provisional’ status according to Refseq (Figure 4A, arrow), but it appears that our experiments have questioned the precision of its annotated genomic location. Alignment results show the NPA transfrag to be homologous to the 5′ region of the human *SNORA61*, whereas the annotated mouse *Snora61* shares a lower homology with the 3′ of the human snoRNA (Supplementary Figure S4A). Northern blots using probes against both the NPA transfrag and the annotated mouse *Snora61* show a single RNA transcript of ∼120 nt, which is more abundant in ESCs than differentiated cell types, and qPCR experiments confirm a significant decrease in expression during differentiation (Supplementary Figure S4B and C).

**Figure 5.**
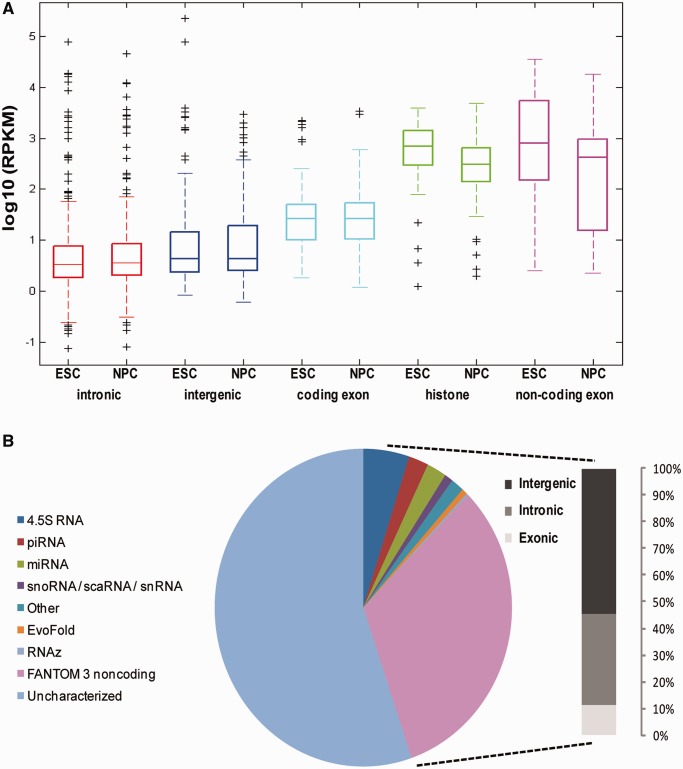
ncRNA genes are highly enriched in the NPA RNA fraction. (**A**) NPA transfrags from ESCs and NPCs were divided according to their overlapping genic type. RPKM scores were calculated based on mapped reads from the NPA RNA-seq experiment. Boxplots represent the distribution of RPKM scores (log scale) for the different categories of NPA transfrags. Outliers are marked with black ‘+’ signs. Transfrags overlapping non-coding exons (pink) and canonical histones (green) have higher RPKM distributions than those overlapping introns (red), exons (light blue) and intergenic regions (dark blue); yet, the many outliers of intronic and intergenic transfrags represent transfrags with RPKM scores comparable with *bona fide* NPA RNA genes. (**B**) BLAST alignment reveals highly significant sequence similarities between NPA transfrags and ncRNA sequences (from www.ncRNA.org), including 4.5S rRNA, piRNA, microRNAs (miRNA), snoRNA, small Cajal body-associated RNA, snRNA and other individual functional ncRNA such as *Rnase P*, *Rmrp* and *Malat-1*. RNA with predicted functional structure (Evofold), structurally conserved and thermodynamically stable RNA (RNAz) and ncRNAs annotated by the FANTOM3 project also share sequence similarity with NPA transfrags. The majority of these similar transfrags are found in Refseq intronic or intergenic regions (right).

As snoRNAs are functional as NPA transcripts, it is not surprising that NPA transfrags should detect them. NPA transfrags overlapped other types of annotated small non-coding RNA (ncRNA) genes, such as *Rmrp* (Figure 4B), *Rpph1* (Figure 4C), *Snord32a*, *Snord35a* and others. Therefore, despite the aforementioned enrichment in overlap of NPA transfrags with protein-coding exons, NPA transfrags can also identify transcription of ncRNAs genes. These genes are generally underrepresented in most expression studies to date, as they are non-coding, incomprehensively annotated and not included in standard microarray expression platforms. We suggest that an additional reason for their underrepresentation is that they are selected against in cases where RNA libraries/samples are generated from only polyA+ RNA and overridden by protein-coding RNA in total RNA samples. We hypothesized that perhaps by selecting against PolyA tails and enriching for NPA RNA, we may be able to better detect such ncRNAs. Indeed, abundance levels in our NPA RNA-seq samples of NPA transfrags overlapping annotated ncRNA genes (nc exon) are even higher than the NPA transfrags in the exonic category, which we examined previously (Figure 5A, pink), confirming that the NPA RNA fraction is enriched with ncRNA. Indeed, most non-coding shortRNAs are NPA, having instead, a well-defined secondary structure or working in complex with other proteins, e.g. snRNA, snoRNA, telomerase. Our control canonical histone gene transfrag set also has higher abundance levels than the (PA) exonic category as expected (Figure 5A, green).

### Detection of novel, small, conserved and non-polyadenylated ncRNA (snacRNA)

It is also apparent that many outliers of the intronic and intergenic transfrags’ RPKM distributions (Figure 5A, plus signs above red and blue boxes) have high RPKM levels similar to transfrags overlapping annotated non-coding (nc exon) and histone genes. This similarity in abundance levels suggests that these outlier intronic and intergenic transfrags could potentially represent *novel* ncRNAs. To assess this, we aligned the sequences of all transfrags from both cell types to the sequences in the Functional RNA Database (fRNAdb) (31), which includes sequences of both reliably and provisionally annotated ncRNAs from mouse and many other species (see ‘Materials and Methods’ section). We found that many transfrags have a highly significant similarity to known ncRNAs from mouse, human and other species showing a high degree of conservation of ncRNA genes (Figure 5B). This includes similarity to ncRNA genes determined by the RIKEN FANTOM3 project (4,49), 4.5S rRNA genes, miRNA precursors, snoRNAs, snRNAs, piRNAs and others. Interestingly, of these functionally characterized transfrags, the majority are currently associated with intronic or intergenic locations vis-à-vis PA protein-coding genes and not yet comprehensively annotated in Refseq (Figure5B, right). The exonic ones are those few examples that are reliably annotated in the mouse genome (nc exon).

**Figure 6.**
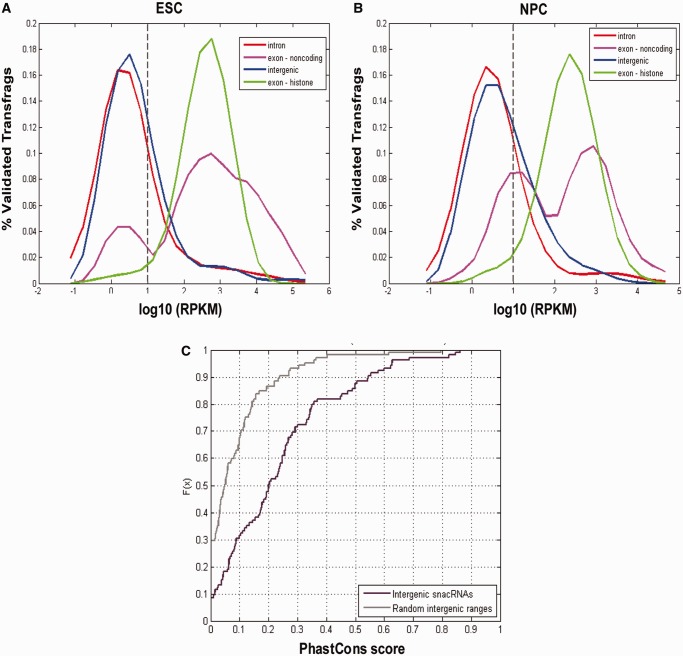
The novel NPA RNAs are highly conserved. (**A** and **B**) NPA transfrags from ESCs (A) and NPCs (B) were divided according to their overlapping genic type. RPKM scores were calculated based on mapped reads from the NPA RNA-seq experiment. Density plots of RPKM scores (log scale) for NPA transfrags according to their genomic region overlap. Both intergenic (blue) and intronic (red) distributions have a substantial right-tail representing transfrags with RPKM levels comparable with non-coding exons (pink) and histone genes (green). Gray dashed line represents minimum RPKM score threshold for identifying snacRNAs. (**C**) Cumulative Distribution Function (CDF) plots of PhastCons conservation score among placental mammals for NPA transfrags with RPKM scores above threshold in both ESCs and NPCs (black) and random range simulation (gray). Only intergenic regions were considered in this analysis to avoid confusion with genic conservation levels. Conservation of these intergenic snacRNAs is highly significant (*P* < 10^−10^, Kolmogorov–Smirnov test).

We defined intronic and intergenic transfrags with the highest potential to be real novel ncRNAs as those with higher RPKM levels than a threshold of 1 in log scale. This threshold represents the fifth percentile of RPKM levels of transfrags overlapping histone gene exons, meaning that by only looking at NPA transfrags above this threshold, we would preserve our 95% recall rate of histone genes (our positive control) (Figure 6A and B). This cutoff was similar for ESCs and NPCs, despite the slight shift (downregulation) of the histone gene abundance distribution; therefore, we chose the mean as a single threshold value for both cell types, which translated into the value of 1 on the log scale of RPKM values, which corresponds to ∼4 mapped reads per 100 nt genomic unit.

We first examined the gene ontology of the genes harboring the intronic ncRNAs above threshold and found a significant enrichment of the terms ‘Translation’, ‘Ribosome’ and ‘Ribonucleoprotein Complex’. These types of genes commonly harbor NPA snoRNAs within their introns (50). Thus, intronic ncRNA transfrags may include novel snoRNAs expressing in ESCs and NPCs, a severely under-annotated group of functional RNAs in mouse. Next, we assessed the conservation levels of the intergenic ncRNAs above threshold, which showed high levels of genomic conservation when compared with random intergenic ranges of the same number and lengths (*P* < 10^−^^10^, Kolmogorov–Smirnov test) (Figure 6C). These same ranges also have low coding potential with only five ranges (<4%), harboring a potential open reading frame of over 100 amino acids. Taken together, these data provide a strong basis for the functional potential of these NPA transfrags as novel ncRNA genes, which we refer to as snacRNAs (short non-polyA conserved RNAs). An example of the high degree of conservation of one snacRNA is shown in Supplementary Figure S5. There are no annotations in this region from Refseq or UCSC gene tracks, and there is no evidence of mouse mRNAs, ESTs or homology to proteins of other organisms, hence the definition of the snacRNA as intergenic and novel. In this case, the ENSEMBL gene prediction track concurs with our prediction and also predicts an ncRNA at the same location on chromosome 7 based on sequence alignment with ncRNA sequences.

### Histone genes and snacRNAs lack standard RNA Polymerase II-associated epigenetic marks

**Figure 7.**
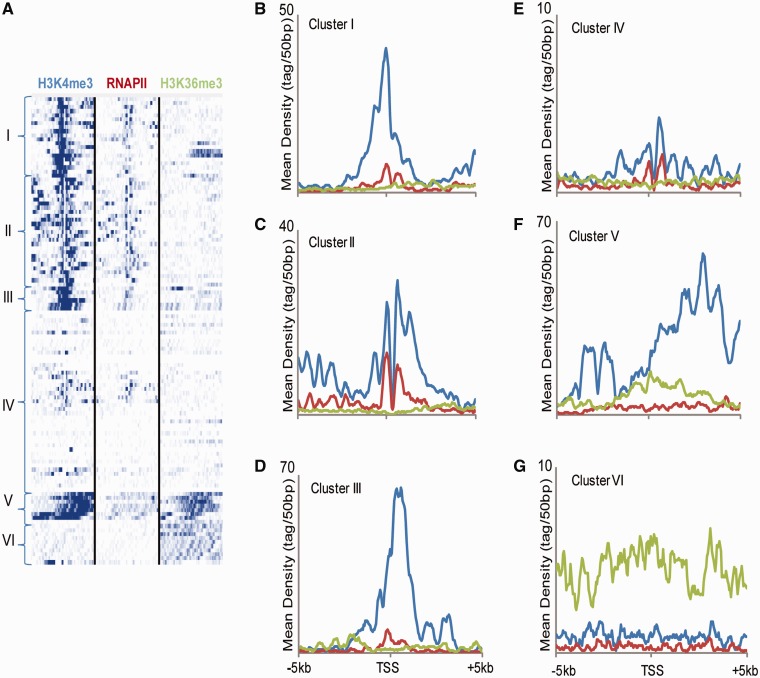
Epigenetic profile of NPA histones and snacRNAs. (**A**) Mapped read densities from ESCs of H3K4me3, RNAPII and H3K36me3 in a window of 10 kb around a set of center-aligned histone genes and intergenic snacRNAs detected in ESCs. K-means clustering and generation of heatmaps were performed using seqMINER. The six major clustered profiles are numbered from I to VI. (**B–G**) Mean mapped read density profiles of H3K4me3 (blue), RNAPII (red) and H3K36me3 (green) in a window of 10 kb around histone gene densities from clusters I to VI, respectively.

We sought to gain insight into the transcriptional regulation properties of the novel, intergenic snacRNAs. The search for RNA polymerase II-associated epigenetic marks, i.e. H3K4me3/H3K36me3 genomic domains, greatly facilitated the discovery of another novel class of functional ncRNAs, termed long-intergenic-ncRNAs (lincRNAs) (51). Yet, lincRNAs are long, PA and undergo splicing, whereas snacRNAs, similar to histone genes, are short, NPA and do not show a multi-exonic pattern. Therefore, their epigenetic pattern could differ from lincRNAs and standard protein-coding genes. To characterize the epigenetic signature of these newly discovered snacRNA transcripts, we analyzed the binding patterns of RNA Polymerase II, H3K4me3, H3K36me3 and other histone modifications at intergenic snacRNA loci as well as histone gene loci as an internal control for RNAPII-mediated NPA transcription, using previously published data sets (36). The chromatin maps were obtained in a different cell line (V6.5) than the one used in this study (R1), but we previously showed that the chromatin landscape is by and large similar between different ES cell lines on a global scale (52). Intronic snacRNAs were excluded from this assay so as not to confuse their epigenetic signature with that of the host genes’ epigenetic patterns. Histone genes show a strong H3K4me3-binding pattern around their transcription start sites (TSS) as expected (Figure 7A I–III). The mean density profiles of histone gene regions show either a single or double ‘K4’ peak around their TSS, characteristic of RNAPII promoter regions (Figure 7B–D). Surprisingly, unlike most RNAPII-transcribed genes, almost no H3K36me3 marks are detected at the TSS or in the gene body of the replication-dependent histone genes. This is in line with a recent observation demonstrating that H3K36me3 is enriched exclusively on spliced exons (53), whereas replication-dependent histones do not undergo splicing.

The intergenic snacRNA transfrags clustered into three distinct epigenetic patterns. The largest cluster included snacRNA regions that had neither K4 nor K36 signal (Figure 7A IV, 7E). We also looked for evidence of other euchromatic and heterochromatic epigenetic marks such as H3K27me3 and H3K9me3, but found no significant enrichment (Supplementary Figure S6A). This may indicate that these RNA genes are regulated and transcribed differently than protein-coding genes. We looked for evidence of Pol III binding (54), which transcribes non-coding and NPA tRNA genes (55), but only found enrichment at a handful of annotated ncRNA genes known to be transcribed by this polymerase (Supplementary Figure S7). Several snacRNA ranges had typical ‘K4-K36’ signature of RNAPII transcribed genes (Figure 7AV and F). These transfrags overlap with exonic regions of the *Malat-1* gene, a lincRNA (a.k.a. *Neat2*), which is transcribed by RNAPII as a 7 kb polaydenylated transcript but undergoes cleavage and processing by RNaseP to produce a shorter NPA transcript, mascRNA (56), leading to its detection as an NPA transfrag in our tiling array analysis. The last group of snacRNAs shows only K36 binding (Figure 7AVI and G). As many of these transfrags are in close proximity to one another, they may represent internal exons of yet un-annotated, protein-coding genes and not *bona fide* ncRNA genes, but this remains to be seen. We performed the same analysis on the NPA transfrags obtained from the NPC sample yielding similar results (Supplementary Figure S6B).

### Potential role of snacRNAs in maintaining stemness and differentiation

**Figure 8.**
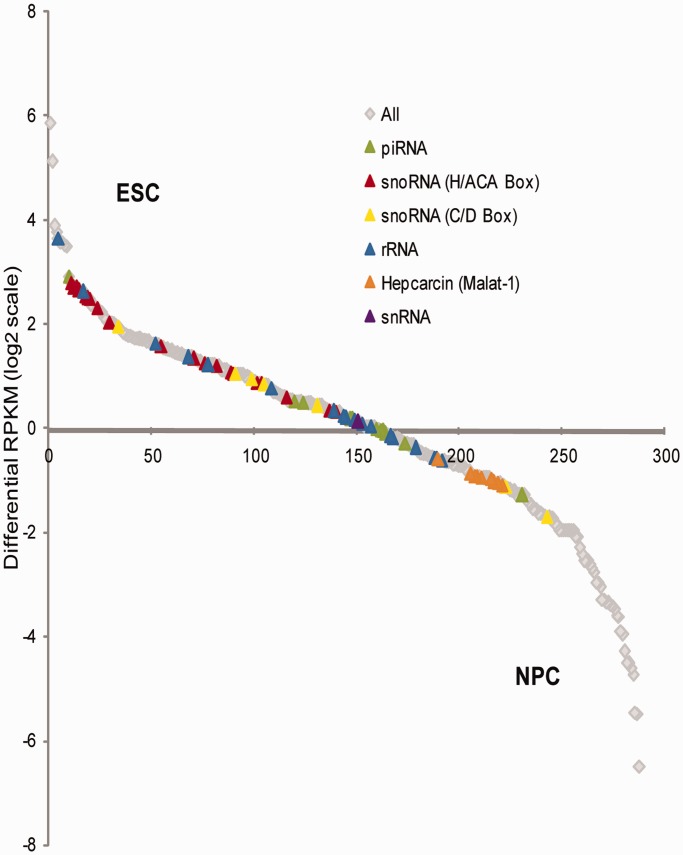
Differential expression of snacRNAs between ESC and NPC. A combined list of snacRNAs and NPA transfrags overlapping annotated ncRNA genes from ESCs and NPCs was generated. Differential RPKM levels between ESC and NPC NPA RNA-seq experiments were measured for each range in the group. Ranges are sorted from highest (ESC enriched) to lowest (NPC enriched) differential RPKM score. All transfrags are depicted by gray diamonds, and Transfrags with fRNAdb characterization (via BLAST alignment) are depicted by color-coded triangles according to legend.

In the case of protein-coding genes, expression level assessment and comparison is a widespread approach for gauging the extent of functional impact in a certain biological context; yet, it is an indirect measurement, as the protein product is subject to post-translational modifications, which may amplify, reduce or even reverse the functional outcome (57). In contrast, non-coding genes are not affected by the regulatory mechanism controlling translation or post-translational modifications, rendering the quantification of RNA abundance levels as a more direct method for measuring functional differences. We, therefore, compared the expression levels of intronic and intergenic snacRNAs and transfrags, which overlapped well-annotated ncRNA genes (nc exon) in ESCs and NPCs. Transfrags overlapping single exon protein-coding histone genes were also included in this analysis. We merged the ESC and NPC transfrag lists so that overlapping ranges are represented only once. Differences in RPKM measurements between ESC and NPC samples were as high as 80-fold corresponding to up to 40% differential read density between cell types (Figure 8). We used the ncRNA categories established by the fRNAdb analysis to identify whether there was any correlation between change in abundance levels and certain known ncRNA subtypes. Interestingly, in contrast to the general trend, most expressing snoRNAs were significantly downregulated on differentiation to NPCs. H/ACA box snoRNAs comprise the bulk of this group, whereas C/D box snoRNAs have more equal numbers of both downregulated and upregulated transcripts. In a recent study of snoRNA expression across 11 human tissues, the authors observed a major shift from C/D box snoRNAs in brain to H/ACA box snoRNAs in testes (58). ESCs were not included in the analysis, but enrichment of H/ACA box snoRNAs in these highly proliferative cells is not surprising, as their main function, pseudouridylation, has a role in ribosome biogenesis, cellular proliferation and pre-mRNA splicing (59). Replication dependent histones are also downregulated on differentiation from ESCs to NPCs (Supplementary Figure S8, Figures 1A, 5A, 6A and B), possibly reflecting the attenuation in proliferation rate. This was also reported by Yang *et al.* when comparing human ESCs to HeLa cells (38). There was no other apparent trend of expression for other major categories of ncRNA, indicating that akin to protein-coding genes, they are versatile in their roles in the scope of stem cell biology. Many of the novel snacRNAs that could not be categorized by sequence homology show large differences in expression among ESCs and NPCs and are good candidates for future functional assays.

### Differential expression of RNA components of the minor spliceosome suggests regulatory role of minor splicing during differentiation

One interesting example of a differentially expressed ncRNA is the snRNA, *U11* (*RNU11*), which was detected by an overlapping NPA transfrag in NPCs, but not in ESCs. *U11* is relatively well characterized as a fundamental RNA component of the minor spliceosomal complex, along with *U12*, *U4atac* and *U6atac*. The minor spliceosome splices out special introns, termed ‘minor’ or ‘U12’ introns, with unique branchpoint sequences and polypyrimidine tracts, and often with non-canonical AT-AC splice sites (60). Approximately 550 genes in the mouse genome contain one or more U12 introns (61) and are generally associated with core housekeeping functions (62,63). We validated this finding with semi-quantitative PCR on both *RNU11* and *RNU12* genes, which shows increased expression of the NPA form of both genes in NPCs (Supplementary Figure S9A and B). The minor spliceosome is considered less efficient than the major one, which perhaps acts as a rate-limiting step in the expression of U12 genes (64); therefore, the differential expression of its components might manifest in changes in splicing efficiencies and/or expression levels of U12 genes. We chose five U12 intron-containing genes from the U12 database (61), which participate in different cellular processes and were characterized as ‘on’ in ESCs and NPCs (see ‘Materials and Methods’ section): *Drap1*, *Psmc4*, *Phb2*, *Gars* and *Eed*. Using qPCR, we found no significant difference in splicing efficiencies of U12 introns, or nearby U2 introns used as internal controls, for four of five of these endogenous genes. However, the polycomb group gene *Eed* (embryonic ectoderm development), which was significantly downregulated in NPCs compared with ESCs (Supplementary Figure S9D), displayed altered splicing efficiency between ESCs and NPCs (Supplementary Figure S9C). The splicing efficiencies of *Eed*’s U12 and U2 control intron were both increased ∼2-fold in NPCs (*P* = 0.02 and 0.06, respectively, Student’s *t*-test). This is particularly interesting, as *Eed* is a component of the PRC2 complex (65), which is important for the maintenance of the stem cell state by mediating developmental gene silencing (66). Although Myc transcription factors have been implicated in the regulation of Polycomb group proteins in stem cells (67), our results may suggest that the *Eed* component may be additionally regulated by the rate-limiting U12 intron excision step.

To assess whether there might be a wider phenomenon of minor splicing regulation affecting gene expression during differentiation, we conducted an expression analysis of all U12 intron containing genes based on microarray gene expression data (68). This analysis revealed no overall trend in U12 gene expression during differentiation, and only 2/5 (<0.3/0.9%) genes were significantly down/upregulated in NPCs relative to ESCs. One of the significantly downregulated genes in this analysis was again *Eed*. It still remains controversial whether levels of minor spliceosomal RNAs have significant effects on U12 gene expression and splicing (30), but this one example portrays how the expression level of one NPA ncRNA can potentially directly and indirectly affect the expression of many differentiation regulators. As NPA ncRNA is involved in such fundamental cellular processes, it cannot be overlooked in comprehensive studies of stem cell biology.

Finally, as a proof of concept for the essential role of some of the ncRNAs, we selected the most highly expressing ncRNA we identified, *Snora61* (Figure 4A) for knockdown analysis. We used 2′-*O*-methyl modified oligos complementary to either *Snora61* or Green Fluorescent Protein (GFP) (69). We found a concentration-dependent decrease in the levels of *Snora61* when we treated the cells with increasing amounts of ASOs, but no effect when cells were treated with ASOs for GFP. Massive cell death was observed in <24 h following the knockdown, whereas control oligos had no discernible effects (Supplementary Figure S10). A similar effect was observed in somatic fibroblasts. This shows that *Snora61* is an essential RNA, although its exact role remains to be investigated.

## DISCUSSION

In this study, we characterized the NPA transcriptome in ESCs and ESC-derived NPCs, by using two complimentary methods for genome-wide transcription analysis. Using a whole mouse genome tiling array, we took advantage of the robustness of the DNA microarray platform, which we increased with the application of threshold criteria separately to three biological repeats. But tiling arrays suffer from cross-hybridization effects and the usage of suboptimal probes, which also introduce false positives; therefore, we also applied RNA-seq to further validate the regions of transcription defined by the array and provide additional data regarding their strand specificity. In all, 30–40% of transfrags contained at least one mapped read, representing 25% of total mapped reads. Similar levels of intersection between the results of the tiling array and the RNA-sequencing data were also perceived in previous studies (24,70) largely owing to the application of stringent selection criteria during data analysis. Therefore, the intersection between the results of the two experimental approaches was used to robustly detect novel NPA transcripts while steering clear of false positives produced by either method. Our cautious approach provides a genome-wide picture of NPA transcription based on a high signal-to-noise ratio.

We find that NPA transfrags overlap many genomic regions throughout all chromosomes, except Y; yet, there is significant enrichment of overlap with exons of protein-coding genes. The overlap with NPA replication-dependent histone genes was found to be, as expected, with a 95% recall rate, but we did not find any apparent common functional role for any other gene subsets overlapped by NPA transfrags. Also, NPA transfrags tended to overlap with only a subset of the exons belonging to a gene with a clear bias toward the 5′-end, suggesting that these are generally not full-length transcripts. This strengthens the conviction that replication-dependent histone genes are the only eukaryotic protein-coding genes with full-length NPA mRNAs (15). We hypothesize that the transfrag analysis detected are either still incomplete, nascent mRNA or semi-degraded mRNA transcripts that should exist in any total RNA sample. The significant association of NPA transfrag overlap with genes with longer transcript length and larger exon count (Supplementary Figure S11) strengthens this claim, as these transcripts would be more likely to take longer to be transcribed or degraded and could be detected by our methodology. Based on the high similarity of the probe intensity profile of the NPA fraction to that of the PA fraction (peaks over exons and valleys over introns), the percentage of nacsent transcription in the NPA fraction is probably minimal, and the 5′ bias is more likely a product of RNA degradation. A similar profile was found by the study of NPA transcription in human HeLa cells by Yang *et al.* (38) who used a similar fractionation protocol, yet a different detection platform, namely RNA-seq. We conclude, that although fractionation by oligo-d(T) to obtain mature mRNAs in the PA fraction is efficient, it does not reciprocally enrich for nascent RNAs in the NPA fraction, as this fraction is too large and diverse, and nacsent RNAs remain only a small and inefficiently detected portion of it.

Yang *et al.* (38) also observed a 5′–3′ gradient of NPA transcription levels over protein-coding genes, but their manual examination did not enable the genome-wide analysis of this phenomenon we found to be prevalent. The intersection between genes harboring NPA exons from our data analysis in mouse and the genes in their data sets in humans (Supplementary Figure S12, Supplementary Table S4) finds mostly canonical histone genes in all cell types as expected from their conservation and ubiquitously expressing nature. A small number of other protein-coding genes exist in that same category (see table). In addition, there is a significant number of common genes to both the hESCs and mESCs and to NPCs and HeLa cells (*P* = 0, permutation test). The level of intersection is also greatly underestimated, as it is done across species. Some protein-coding genes are common to other previous studies of protein-coding genes with short polyA tails such as *Actin-b* (39). These observations indicate that the NPA transcription we observed for known protein-coding genes is not random, but a biologically regulated property. Further experimentation is required to discern whether these transcripts are nascent transcription, semi-degraded mature mRNAs, splicing intermediates or full-length mRNAs with short polyA tails. Differential regulation of these processes likely exists between undifferentiated and differentiated cell types, as NPA transcriptional behavior cannot be explained as a byproduct of open chromatin in ESCs, as it was found less prevalent in this cell type in both Yang *et al.* (38) and our studies.

In concordance with Yang *et al.*, we also find higher levels of histone gene expression in ESCs versus the differentiated cell type (Figures 1A, 5A, 6A and B); but in our case, we tested ESC-derived NPCs, an isogenic and karyotypically normal cell type. Conversely, we found no evidence of histone gene specificity to ESC type, as all canonical histone genes had overlapping transfrags and mapped reads in both cell types. Higher histone levels shown across species could be attributed to the unique and open chromatin structure in mouse and human ESCs, but may also be attributed to the rapid cell-cycle, comprised largely of S-phase, in ESCs. Regardless of expression level and cell type, histone marks deviate from the classical RNAP II/K4/K36 signature. Instead, we find low levels of H3K36me3 in gene body and vicinity. H3K36me3 has been implicated in the exon–intron architecture of genes and in the crosstalk between transcription and splicing (71,72). Its absence at RNAPII transcribed histone genes, which contain only a single exon and do not undergo RNA splicing, is another strong piece of evidence for this hypothesis (53).

Despite the enrichment at protein-coding exons, we discovered that NPA RNA transfrags overlap many annotated ncRNA genes. These RNAs have a wide range of functions involved in the regulation and execution of transcription and translation processes; yet, they all share a similar feature—a NPA 3′-end. Some of them fold into distinct functional secondary structures, whereas others work in complex with other RNA and protein components. It is possible that a polyA tail would interfere with structure formation or interaction with proteins such that it is excluded specifically from functional ncRNAs, in sharp contrast to its essentiality in stabilizing protein-coding mRNAs.

By combining microarray and sequencing data and focusing on intergenic and intronic regions, we compiled a list of novel snacRNAs, which show low coding potential and high conservation, suggesting that they are likely functional as RNA transcripts. Substantial differential expression of snacRNAs between ESCs and NPCs suggests that they may play critical functional roles in stem cell biology. As translational regulation is irrelevant for ncRNAs, this measure of differential expression is an even more direct measurement of differential functionality than standard protein-coding gene expression arrays.

In this manner, we detected that *U11*, a minor spliceosome core component, is upregulated in NPCs. By applying standard functional assays to assess the impact of this phenomenon, we discovered that the minor spliceosome may play a role in the regulation of a key PRC2 component, *Eed*, which in turn impacts the expression of many developmental genes in ESCs and during differentiation. The effects of the changes in *U11* expression appear to be limited to *Eed* and few other genes, as no overall significant differences in expression or splicing efficiency of U12 intron-containing genes were measured in both qPCR and bioinformatic analysis. The role of minor splicing in gene regulation is not entirely established with most studies performed on exogenous mini-gene constructs, but mutations in the *U4atac* ncRNA component have surfaced in recent publications (73,74), as causal mutations in the severe developmental disorder, Microcephalicosteodysplastic primordial dwarfism, type 1, manifested in post-natal growth retardation and infant mortality. More critical analysis of the downstream effects of minor spliceosome depletion is required to discern its role in stem cell biology and early development.

Although some annotated ncRNAs have well-documented function such as tRNAs and rRNAs, or partially discerned function such as minor spliceosome components, novel ncRNA families are harder to characterize. Yet, there is growing evidence that these ncRNAs play crucial roles in many fundamental cellular processes. LincRNAs, for example, which were discovered in 2009, have already been functionally characterized in recent years and found to carry out significant functional roles involved in gene regulation (75,76), chromatin structure (77), pluripotency (78) and reprogramming (79). Still, the lack of knowledge and functional assays to test ncRNA function impedes the functional characterization of snacRNAs and even the assessment of the extent that annotated ncRNA influences stem cell biology. Future work will need to address other functional aspects of these RNAs such as structure and association with protein to get a better understanding of their functional roles and importance.

Curiously, the trend of more pervasive NPA transcription is apparent starting from the tiling array analysis and persisting through the RNA-seq validations even with different thresholding criteria. The trend is stable even when looking at subcategories of the genic territories where NPA transfrags reside: overlapping exons or introns of annotated protein coding genes, as well as the intergenic regions between them and even the overlap with annotated ncRNA genes (Supplementary Table S5). The biological importance of this trend, which contrasts the ESC pervasive transcription paradigm (based on PA transcription) is still not clear. We predict that the functional characterizations of new ncRNA groups such as snacRNAs as well as better characterization of RNA biogenesis and processing properties will shed light on this observation and the importance of its impact on stem cell biology.

## SUPPLEMENTARY DATA

Supplementary Data are available at NAR Online: Supplementary Tables 1–5, Supplementary Figures 1–13 and Supplementary File 1.

## FUNDING

Funding for open access charge: Israel Science Foundation [ISF 1252/12 and 657/12 to E.M.]; the Israel Ministry of Science (infrastructure grant to E.M.); the Abisch-Frenkel Foundation (to E.M.) and the European Research Council [ERC-281781 to E.M.].

*Conflict of interest statement*. None declared.

## Supplementary Material

Supplementary Data
