# Supplementary material for: Non-polyadenylated transcription in embryonic stem cells reveals novel non-coding RNA related to pluripotency and differentiation
Source: Nucleic Acids Res. 2013 Apr 27;41(12):6300–15. doi: 10.1093/nar/gkt316 (PMC3695530; doi:10.1093/nar/gkt316)
Supplement: Supplementary Data [file supp_41_12_6300__index.html]

Non-polyadenylated transcription in embryonic stem cells reveals novel non-coding RNA related to pluripotency and differentiation — Non-polyadenylated transcription in embryonic stem cells reveals novel non-coding RNA related to pluripotency and differentiation — Supplementary Data 

# Non-polyadenylated transcription in embryonic stem cells reveals novel non-coding RNA related to pluripotency and differentiation

## Supplementary Data

files

**Files in this Data Supplement:**

- Supplementary Data - zip file
