# Supplementary material for: Non-polyadenylated transcription in embryonic stem cells reveals novel non-coding RNA related to pluripotency and differentiation
Source: Nucleic Acids Res. 2013 Apr 27;41(12):6300–15. doi: 10.1093/nar/gkt316 (PMC3695530; doi:10.1093/nar/gkt316)
Supplement: Supplementary Data [file supp_gkt316_suppl_data.zip › nar-00219-v-2013-File010.pdf]

Supplementary Figures

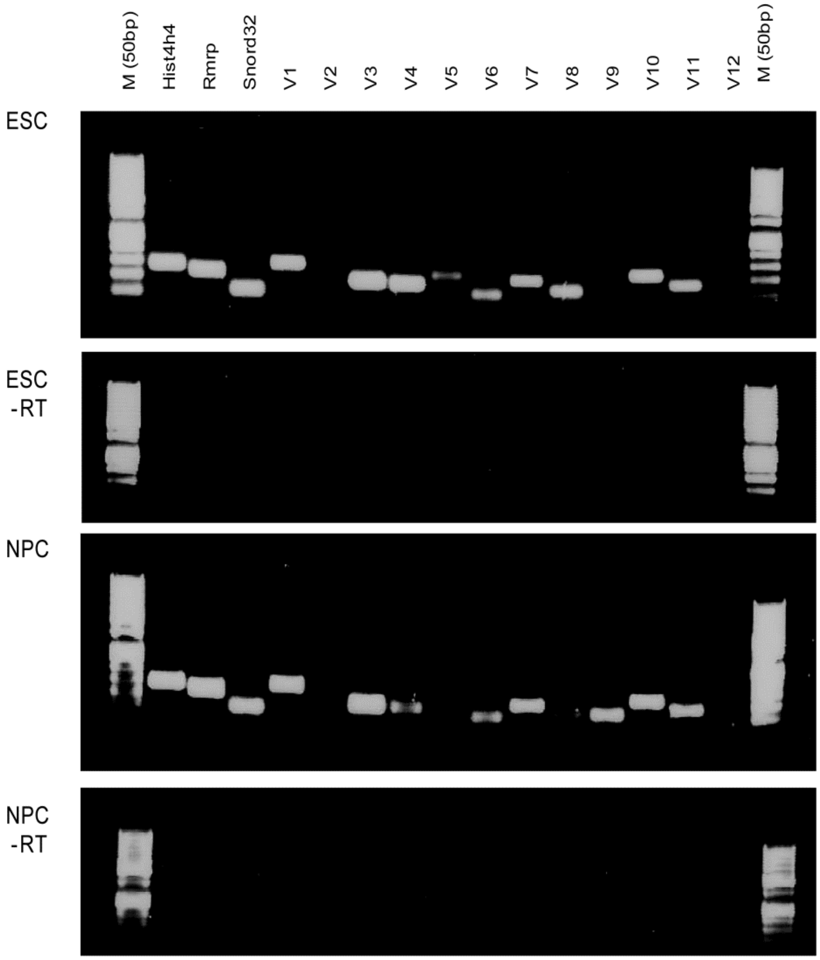

Figure S1

Supplementary Figure S1. Validation of NPA transfrags

RT-PCR of NPA transfrags in ESCs (top 2 panels) and NPCs (bottom 2 panels). All products had the appropriate size on ethidium bromide stained agarose gel (2% Agarose, 1x TAE, 50 bp ladder). NPA transfrags that overlapped with annotated NPA genes were selected as positive controls and are depicted by gene symbol. NPA transfrags that were randomly selected for verification are depicted by V1-V12. Negative controls without RT enzyme are shown in panels 2 and 4 for ESCs and NPCs, respectively.

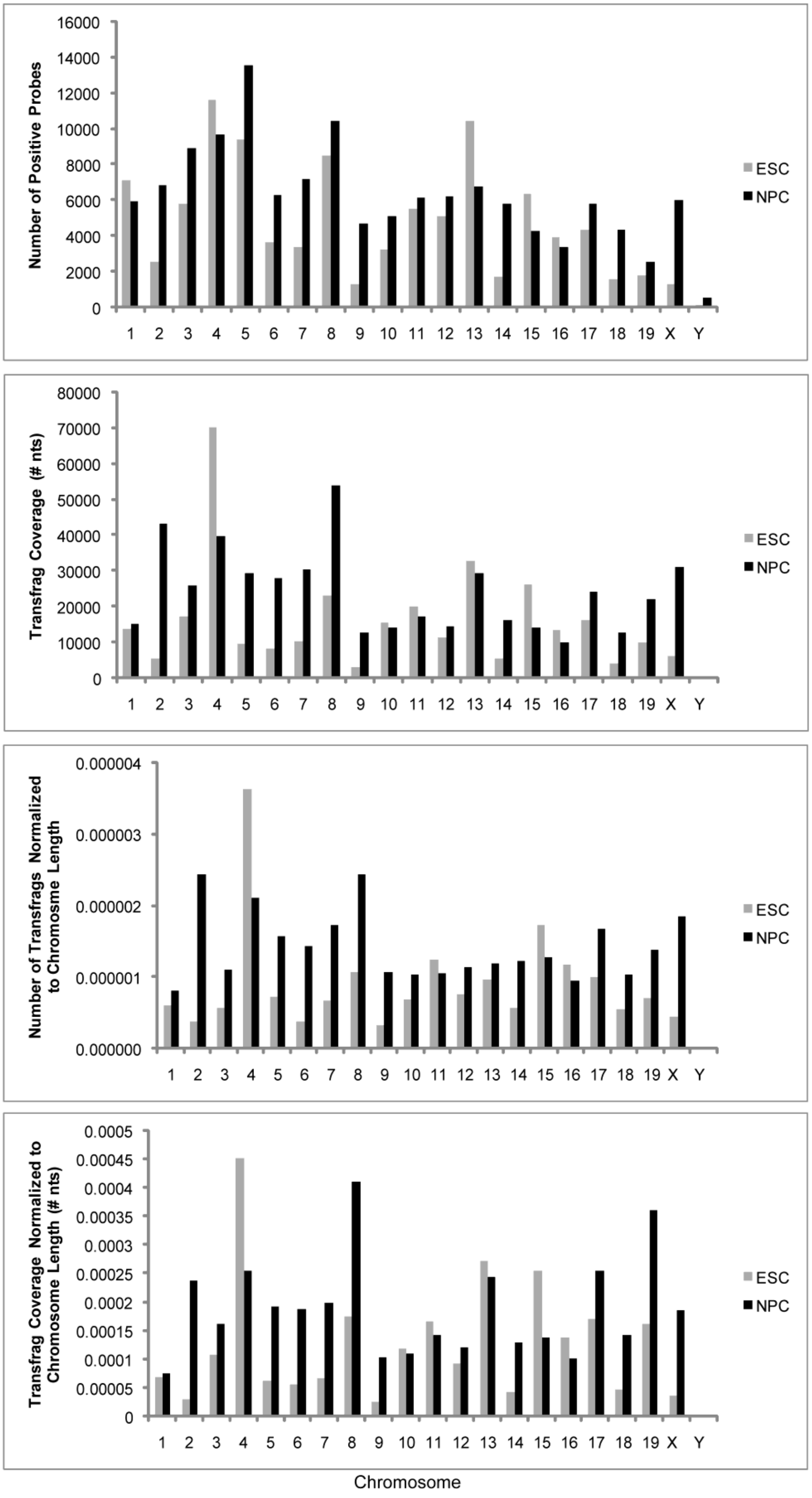
Number of Positive Probes
16000
14000
12000
10000
8000
6000
4000
2000
0
1 2 3 4 5 6 7 8 9 10 11 12 13 14 15 16 17 18 19 X Y
ESC
NPC
Transfrag Coverage (# nts)
80000
70000
60000
50000
40000
30000
20000
10000
0
1 2 3 4 5 6 7 8 9 10 11 12 13 14 15 16 17 18 19 X Y
ESC
NPC
Number of Transfrags Normalized to Chromosme Length
0.000004
0.000003
0.000002
0.000001
0.000000
1 2 3 4 5 6 7 8 9 10 11 12 13 14 15 16 17 18 19 X Y
ESC
NPC
Transfrag Coverage Normalized to Chromosome Length (# nts)
0.0005
0.00045
0.0004
0.00035
0.0003
0.00025
0.0002
0.00015
0.0001
0.00005
0
1 2 3 4 5 6 7 8 9 10 11 12 13 14 15 16 17 18 19 X Y
ESC
NPC
Chromosome

Figure S2

Supplementary Figure S2. NPA transcription is more prevalent in NPC than ESC.

NPA transcription in ESC and NPC samples across mouse chromosomes as depicted by 1) positive probes 2) number of transfrags normalized to chromosome length 3) transfrag coverage (sum of all transfrags' lengths) 4) transfrag coverage normalized to chromosome length. Positive probes are those above noise threshold with NPA/PA intensity ratio ≥ 1. They represent a measure of NPA transcription before transfrag analysis. Chromosome length was extracted from the UCSC chromInfo table.

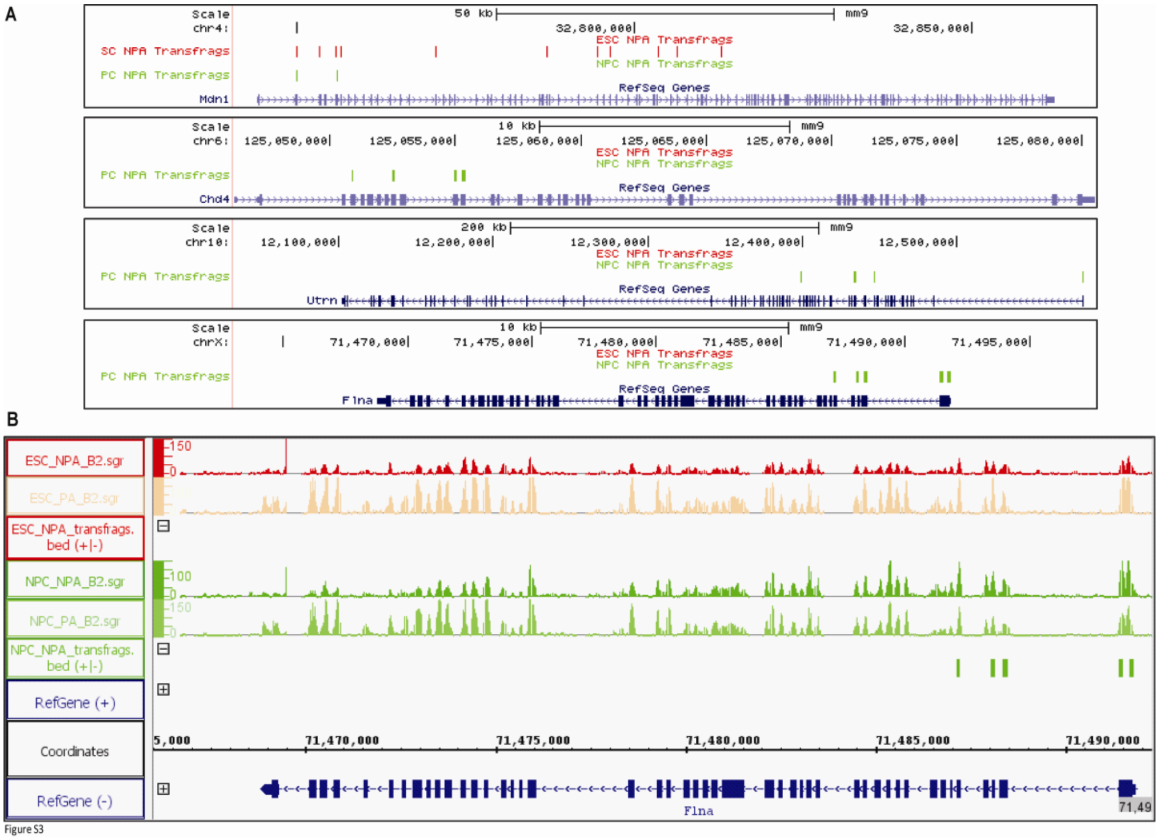

Supplementary Figure S3: Examples of bias of NPA transfrag overlap with 5' exons of protein-coding genes.

(**A**) Schematic view of chromosomal location of NPA transfrags from ESCs (red) and NPCs (green) overlapping 4 gene regions of protein-coding genes: *Mdn1*, *Chd4*, *Utrn* and *Flna*. To

generate these figures ESC NPA transfrags and NPC NPA transfrags were uploaded as custom tracks to UCSC Genome Browser. Details on how to generate a similar session with the data can be found in Supplementary File 1.

(**B**) Normalized tiling array probe signal intensities (on an arbitrary scale of 0-150) along the genomic region on chrX of the *Flna* gene. The polyadenylated (PA) and non-polyadenylated (NPA) RNA fractions from ESCs (top, reds) and NPCs (middle, greens) are shown. The NPA transfrags compiled from the tiling array probe data are shown in similar color scheme. In this example, only the NPC sample contains NPA transfrags (green boxes), which are overlapping exons located at the 5' end of the Flna gene as indicated by the arrows on the gene annotation. Peaks in probe intensities are also highly correlated with gene exons and not introns. The Integrated Genome Browser (IGB) was used to generate this figure and .sgr and .bed files can be found at http://meshorerlab.huji.ac.il/resources/NPA-RNA.

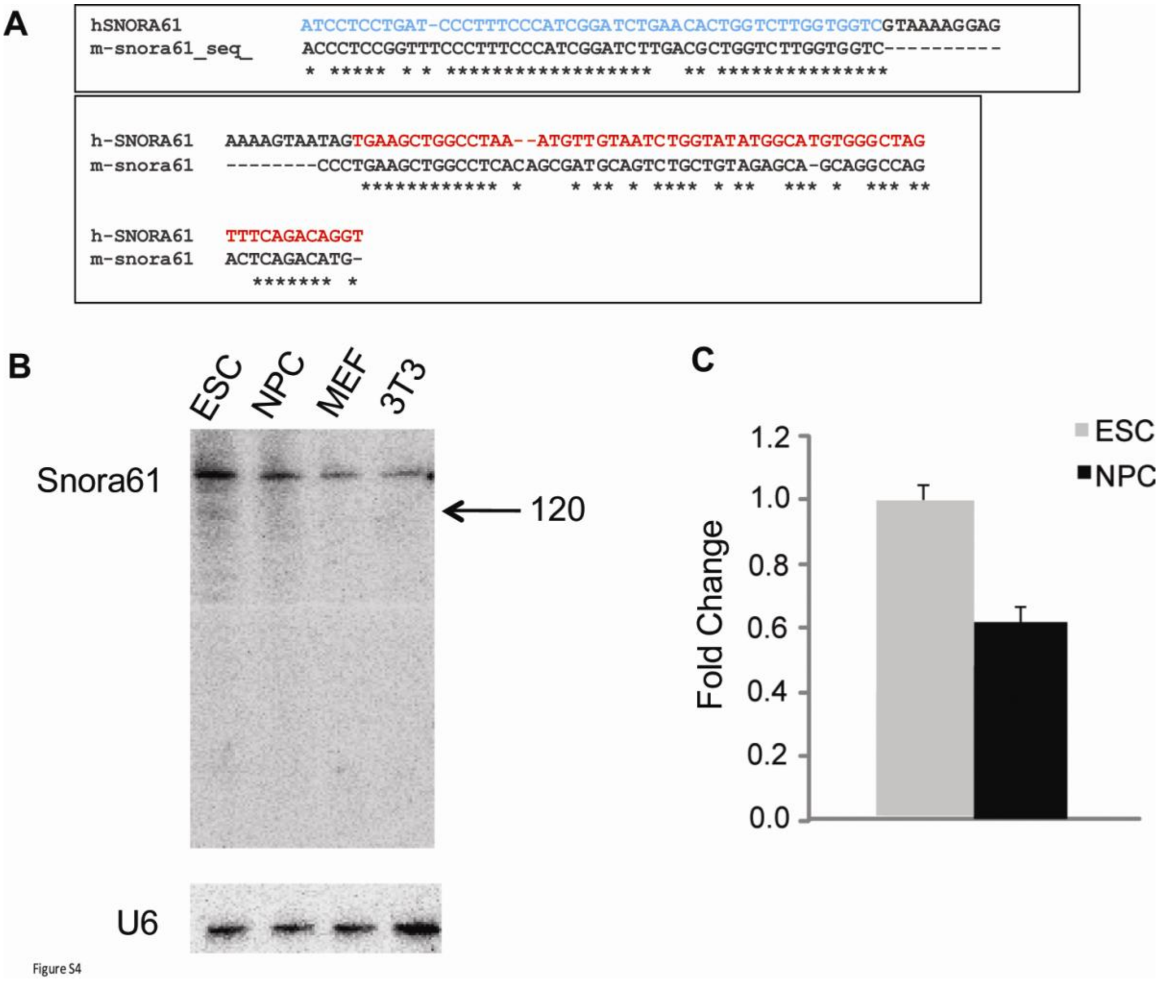

Supplementary Figure S4: Snora61 annotation correction.

(**A**) Blast alignment of the NPA transfrag (m-snora61_seq_) upstream of annotated mouse *Snora61* gene to the 5' end of human SNORA61 gene (top box) and the annotated mouse *Snora61* itself (m-snora61) to the 3' end of the human snoRNA (bottom box). **(B)** Northern blot with probe against m-snora61_seq_ in ESCs, NPCs, MEFs and 3T3 cells shows a single ~120nt product in all cell types. *U6* probe is used as loading control. (**C**) qPCR of m-snora61_seq_ NPA transfrag in ESC and NPC shows ~40% decrease in expression during differentiation.

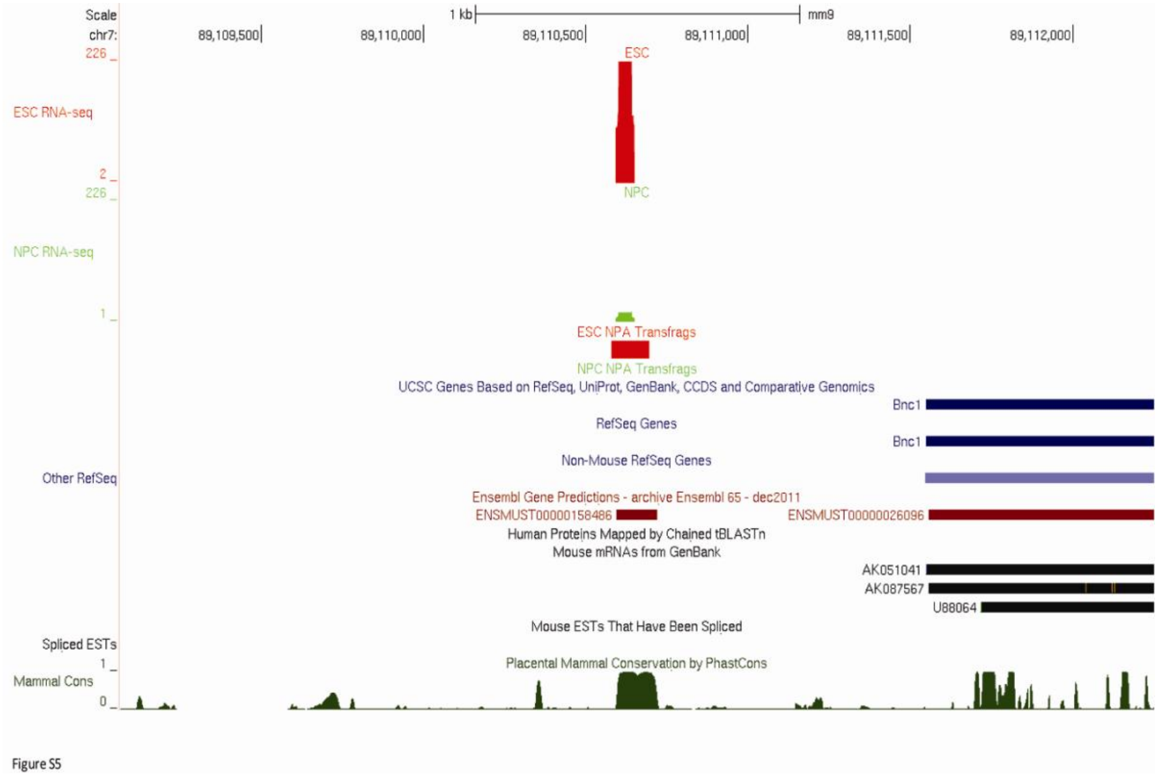

Supplemental Figure S5: Example of high conservation of novel snacRNA.

Genomic region of novel snacRNA on chromosome 7 of mm9 annotation in UCSC Genome Browser. Custom tracks for NPA RNA-seq mapped reads and NPA transfrags in ESCs (red) and NPCSs (green) show detection of NPA transcription from ESC sample in the intergenic space next to the *Bnc1* gene. Neither the Refseq Genes and UCSC Genes (top, blues) nor the mRNA and EST tracks (bottom, black) have any evidence of gene annotation in this region. Also, no human proteins share homology with this region (Human Proteins Mapped by Chained tBlastn track) and there is no documented homology to Refseq genes from other organisms as indicated by the Non-Mouse Refseq Genes track. The ENSEMBL gene prediction track contains a prediction of a non-coding RNA at the same location as the NPA transfrag, which is observably highly conserved (bottom, dark green).

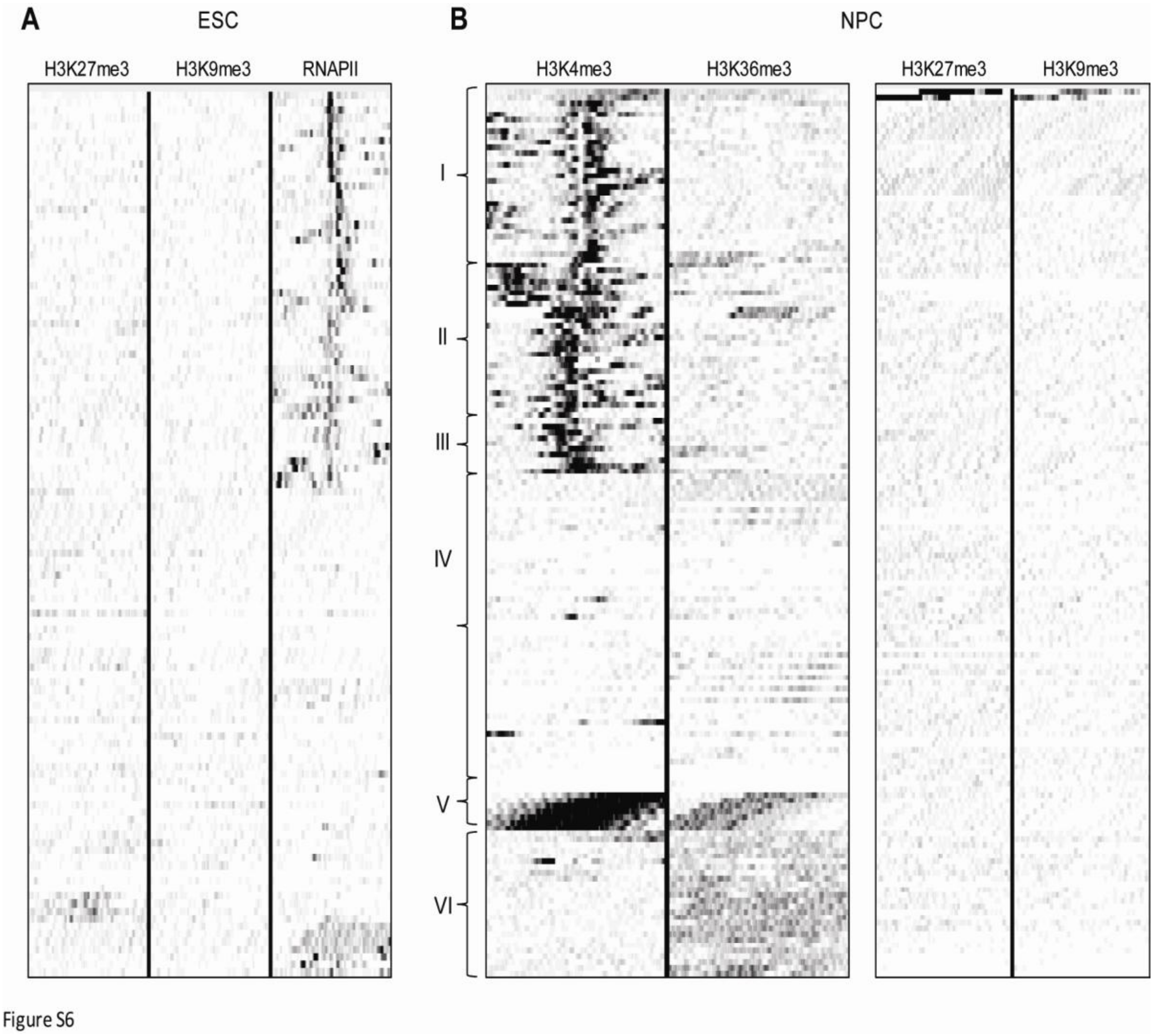

Supplementary Figure S6. Epigenetic profile of non-polyadenylated histone and snacRNA genes.

(**A**) Mapped read densities from ESC sample of H3K27me3, H3K9me3, and RNAPII in a window of 10 kb around a set of center-aligned histone genes and snacRNAs shows no enrichment of these histone modifications. (**B**) Mapped read densities from NPC sample of H3K4me3 and H3K36me3 (left) and H3k27me3 and H3k9me3 (right) in a window of 10 kb around a set of center-aligned histone genes and snacRNAs. K-means clustering and generation of heatmap were performed using seqMINER 1.2. The 6 major epigenetic profiles resulting from clustering the H3K4me3 and H3K36me3 read densities are numbered from I-VI (see Figure 7).

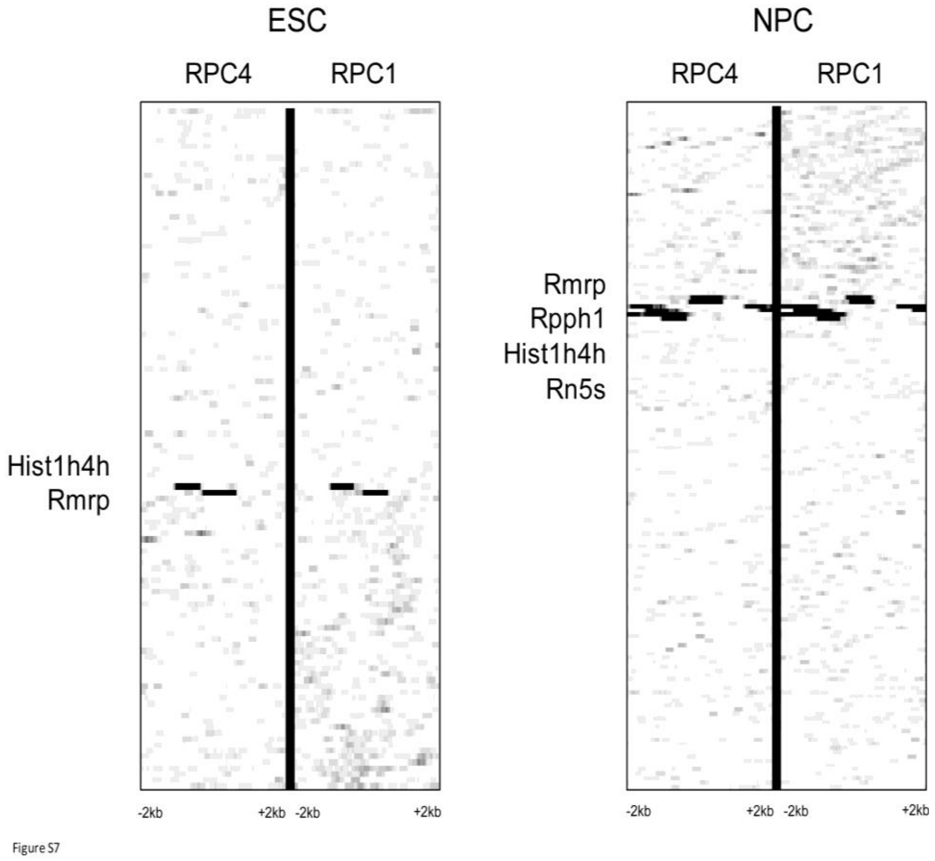

Supplementary Figure S7: RNA Polymerase III distribution at snacRNA sites.

Binding densities of RNAPIII subunits, *RPC1*, and *RPC4*, in the 2kb vicinity of intergenic snacRNAs and histone genes found in ESCs (left) and NPCs (right) respectively. Regions with higher binding densities (black clusters) are depicted by overlapping gene name. *Rmrp* and *Rpph1* are known Pol III transcripts. *Hist1h4h* is ~0.5 kb downstream to a predicted tRNA transcript. *Rn5S* is a predicted 5S ribsomal precursor RNA.

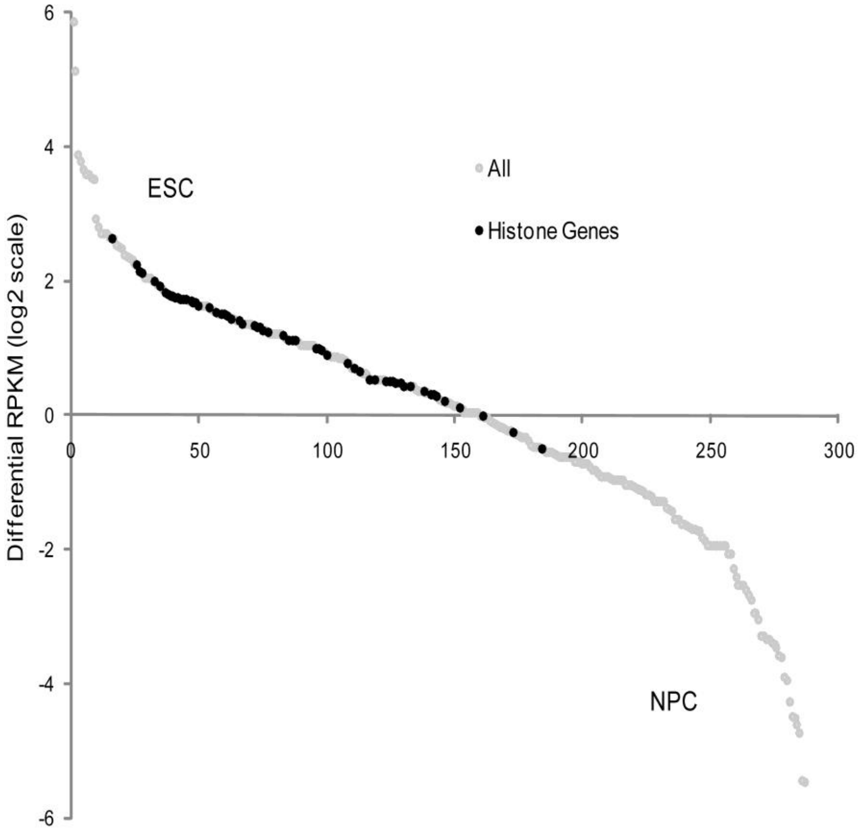

Figure S8

Supplementary Figure S8. Differential expression of NPA transfrags overlapping histone genes shows enrichment in ESCs.

Differential RPKM values (log scale) per histone gene sorted from highest (ESC enriched) to lowest (NPC enriched).

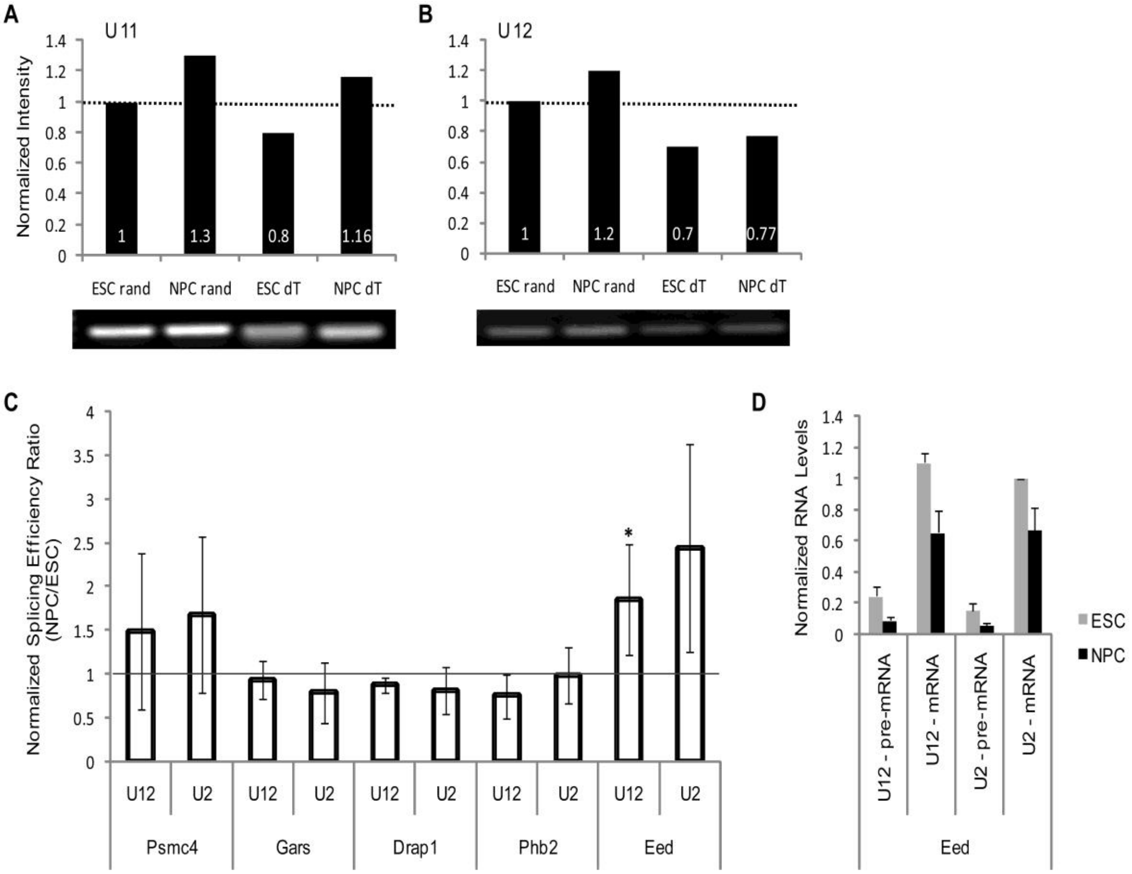

Figure S9

Supplementary Figure S9. Functional analysis of the minor spliceosome in ESCs and NPCs

(**A-B**) Semi-quantitative RT-PCR of *RNU11* and *RNU11* genes in ESCs and NPCs, respectively, shows higher expression levels in NPCs. Reverse transcription with random primers shows a higher signal for both U11 and U12 snRNAs than the oligo(dT) primers indicating enrichment of non-polyadenylated RNA forms of these genes. (**C**) Normalized splicing efficiency ratios (NPC/ESC) of 5 U12 intron-containing genes. Splicing efficiencies were measure as the ratio between mRNA to pre-mRNA product as measured by primers around the U12 intron or nearby internal U2 intron control region. *Eed* showed significant increase in splicing efficiency in NPCs ($p < 0.02$, Student's t-test). Error bars depict standard deviations of triplicate measurements.

(**D**) Normalized RNA levels of Eed mRNA and pre-mRNA as measured around the U12 intron and nearby internal U2 intron. U2 mRNA level in ESCs was set to 1 and the rest were normalized against it, to show a significant down regulation of *Eed* expression in NPCs compared to ESCs. Error bars depict standard deviations of triplicate measurements.

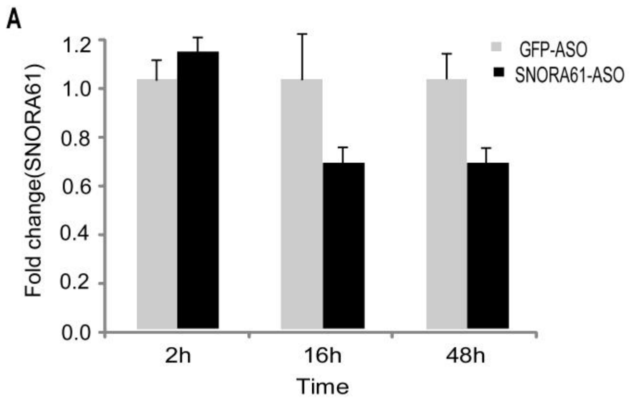

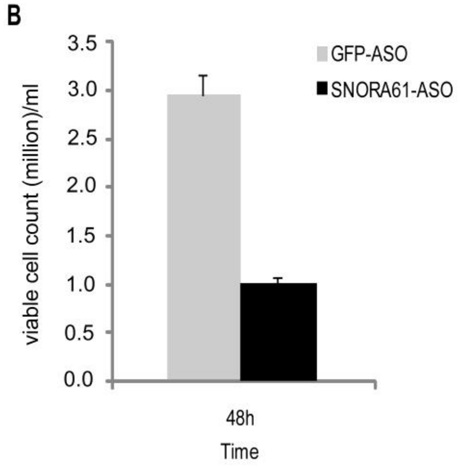

Figure S10

Supplementary Figure S10: SNORA61 knockdown results in decreased cell viability.

(**A**) *SNORA61* knockdown levels by SNORA61-ASO (2-*O*-methylated antisense oligonucleotides) in R1 ESCs. The figure shows expression levels of *SNORA61* in R1-ESCs before knockdown and after 16hrs and 48hrs post transfection. (**B**) Effect of *SNORA61* knockdown on cell viability at 48hrs post transfection.

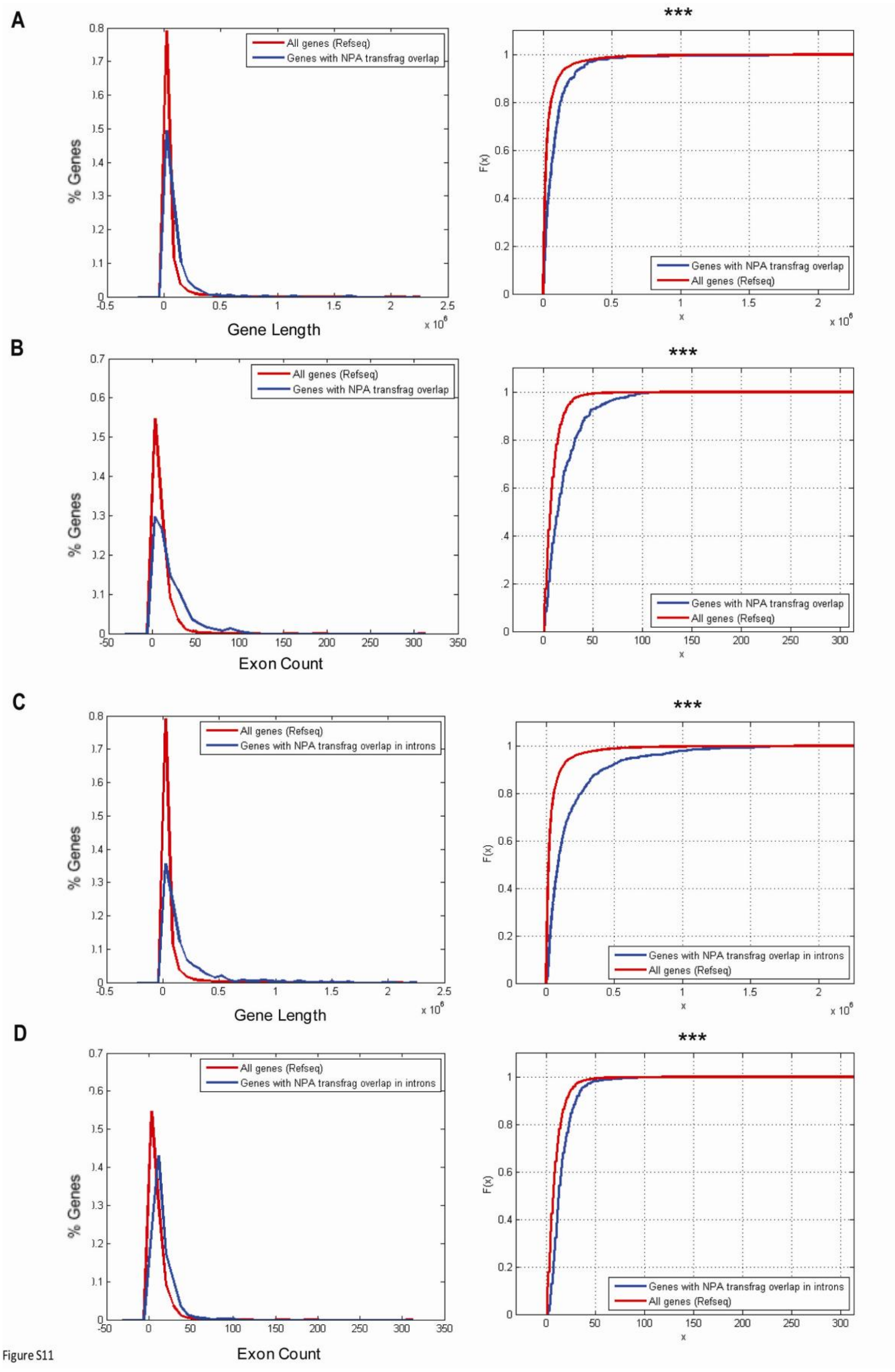
A
B
C
D
***
All genes (Refseq)
Genes with NPA transfrag overlap
Genes with NPA transfrag overlap in introns
% Genes
Gene Length
Exon Count
F(x)
x
x 10^6

Figure S11

Supplementary Figure S11: Genic NPA transfrags are associated with longer transcripts.

Gene length and exon count distributions of genes containing NPA transfrags within their exons (**A-B**) and introns (**C-D**) (blue) compared to the background distribution of all genes (red). Cumulative distribution plots (CDF) on the right depict the overall difference between the distributions, which are all statistically significant (Kolmogorov-Smirnov test, $p< 10^{-10}$), marked with '***'.

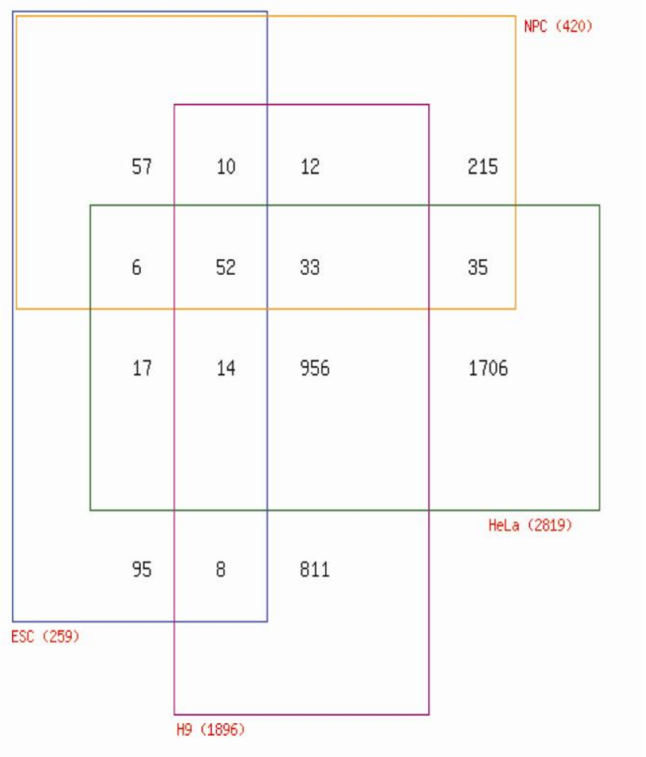

Figure S12

Supplementary Figure S12. Intersection analysis of NPA genes from this paper with previous work done by Yang et al.

4-way Venn diagram of intersections between genes found in ESCs and NPCs to include some region of NPA transcription in this analysis (overlapping NPA transfrags) and genes termed NPA or "bimorphic" according to Yang. et al.'s study on human ESC and HeLa cells. Total numbers of

genes examined are in red parenthesis next to each relevant cell type. Black numbers depict number of genes that are common to the intersecting groups. Gene intersection was calculated based on gene names.

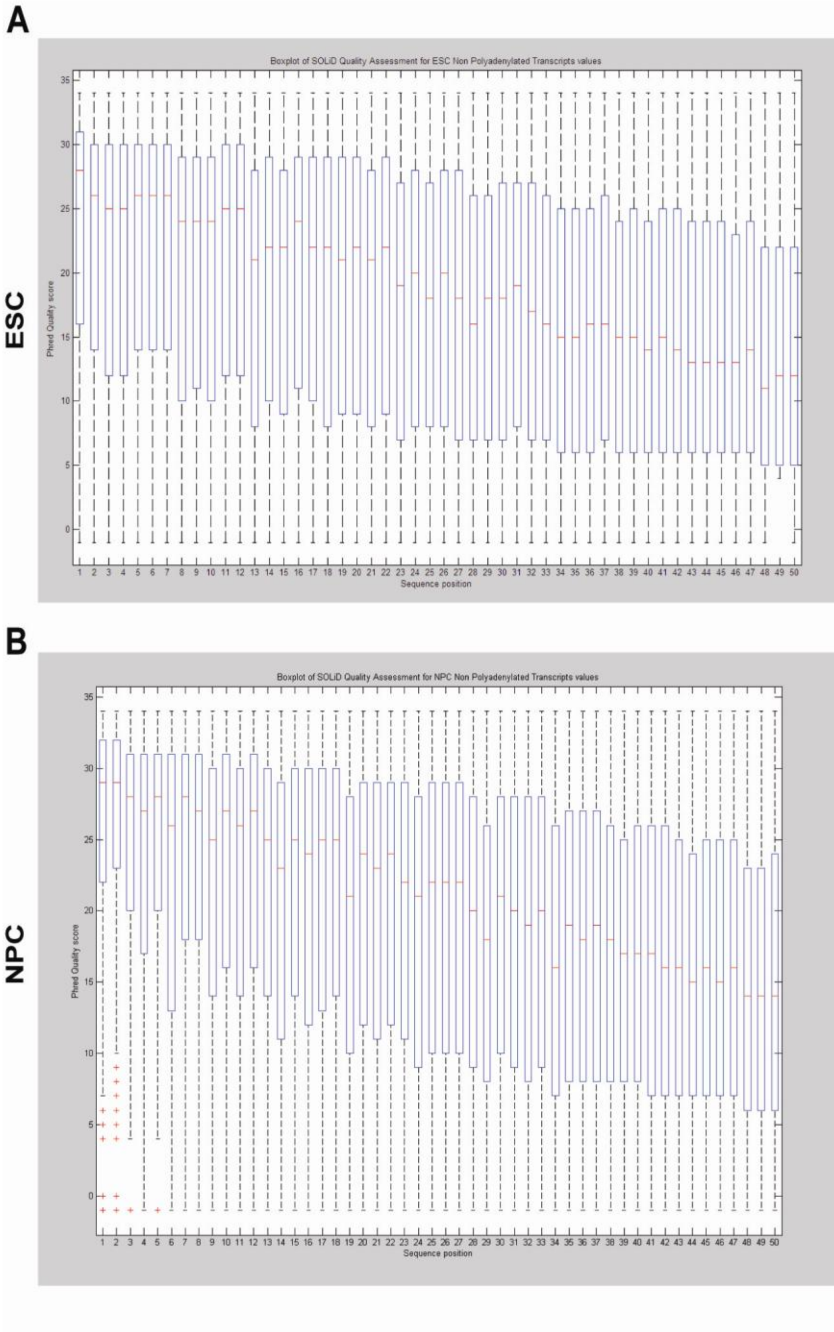

Figure S13

Supplementary Figure S13: Boxplot quality scores of the first 1 million reads in ESCs (A) and NPCs (B).
